# Blood Milieu in Acute Myocardial Infarction Reprograms Human Macrophages for Trauma Repair

**DOI:** 10.1002/advs.202203053

**Published:** 2022-12-16

**Authors:** Margaux A. C. Fontaine, Han Jin, Mick Gagliardi, Mat Rousch, Erwin Wijnands, Monika Stoll, Xiaofei Li, Leon Schurgers, Chris Reutelingsperger, Casper Schalkwijk, Nynke M. S. van den Akker, Daniel G.M. Molin, Lars Gullestad, Jan Eritsland, Pavel Hoffman, Mona Skjelland, Geir Ø. Andersen, Pål Aukrust, Joël Karel, Evgueni Smirnov, Bente Halvorsen, Lieve Temmerman, Erik A.L. Biessen

**Affiliations:** ^1^ Cardiovascular Research Institute Maastricht University Medical Center (CARIM) Maastricht University Maastricht The Netherlands; ^2^ Department of Pathology Maastricht University Maastricht The Netherlands; ^3^ Department of Physiology Maastricht University Maastricht The Netherlands; ^4^ Institut für Humangenetik Westfälische Wilhelms‐Universität Münster Münster Germany; ^5^ Department of Biochemistry Maastricht University Maastricht The Netherlands; ^6^ Department of Internal Medicine Maastricht University Maastricht The Netherlands; ^7^ Department of Cardiology Oslo University Hospital Rikshospitalet Oslo Norway; ^8^ K. G. Jebsen Cardiac Research Centre and Centre for Heart Failure Research University of Oslo Oslo Norway; ^9^ Department of Cardiology Oslo University Hospital Ullevål University of Oslo Oslo Norway; ^10^ Research Institute of Internal Medicine Oslo University Hospital Rikshospitalet University of Oslo Oslo Norway; ^11^ Department of Neurology Oslo University Hospital Rikshospitalet University of Oslo Oslo Norway; ^12^ Section of Clinical Immunology and Infectious Diseases Oslo University Hospital Rikshospitalet University of Oslo Oslo Norway; ^13^ Department of Data Science and Knowledge Engineering (DKE) Maastricht University Maastricht the Netherlands; ^14^ Institute for Molecular Cardiovascular Research (IMCAR) Universitätsklinikum Aachen Aachen Germany

**Keywords:** acute myocardial infarction, heart failure, inflammation, macrophage reprogramming, plasticity, repair

## Abstract

Acute myocardial infarction (AMI) is accompanied by a systemic trauma response that impacts the whole body, including blood. This study addresses whether macrophages, key players in trauma repair, sense and respond to these changes. For this, healthy human monocyte‐derived macrophages are exposed to 20% human AMI (*n* = 50) or control (*n* = 20) serum and analyzed by transcriptional and multiparameter functional screening followed by network‐guided data interpretation and drug repurposing. Results are validated in an independent cohort at functional level (*n* = 47 AMI, *n* = 25 control) and in a public dataset. AMI serum exposure results in an overt AMI signature, enriched in debris cleaning, mitosis, and immune pathways. Moreover, gene networks associated with AMI and with poor clinical prognosis in AMI are identified. Network‐guided drug screening on the latter unveils prostaglandin E2 (PGE2) signaling as target for clinical intervention in detrimental macrophage imprinting during AMI trauma healing. The results demonstrate pronounced context‐induced macrophage reprogramming by the AMI systemic environment, to a degree decisive for patient prognosis. This offers new opportunities for targeted intervention and optimized cardiovascular disease risk management.

## Graphical abstract

A heart infarct induces profound changes to blood composition, which may impact key cells in repair of the heart after infarction: the macrophages. Here, this work demonstrates that macrophages are reprogrammed by their exposure to blood from infarcted patients. This has consequences for the patients’ future cardiovascular health but also offers new opportunities for therapeutic intervention.

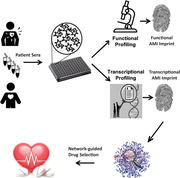

## 1 Introduction

Acute myocardial infarction (AMI) elicits an immediate trauma response with major repercussions for blood composition, including leukocytes and plasma proteome.^[^ ^1^ ^]^ While this response is decisive for proper infarct healing, it may also affect distal atherosclerosis, increasing the risk of a secondary event.^[^ ^2^ ^]^ Recovery of heart function after infarct is controlled by the cardiac macrophage pool.^[^ ^3^ ^]^ During the first 24 h, cardiac‐resident macrophages in the infarct zone will be supplemented by recruited, mainly spleen‐derived monocytes, to assist in debris clearance, reperfusion damage control, and tissue repair.^[^ ^4^ ^]^ Simultaneously, this first inflammatory phase is characterized by considerable vascular leakage and myocardial edema.^[^ ^5^ ^]^ Considering that macrophages are highly plastic cells that adopt a wide variety of effector phenotypes in response to environmental challenges,^[^ ^6^ ^]^ we hypothesized that the altered AMI patient environment will impact on macrophage functionality and thereby on recovery of cardiac function and cardiovascular health in the aftermath of AMI. Here, we present evidence that the AMI systemic environment transcriptionally and functionally reprograms human macrophages for improved clearance, with potentially profound impact on disease outcome. We validated this imprinting in an independent patient cohort and, at transcriptional level, in an open access cohort. Moreover, we identified a gene program that associated with poor patient prognosis and identified candidate drugs able to rescue adverse macrophage programming.

## 2 Results

### 2.1 AMI Serum Exposure Elicits a Distinct Transcriptional and Functional Signature in Primary Human Macrophages

**Figure 1:**
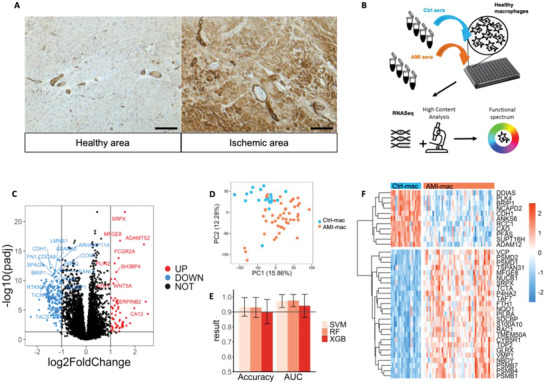
Acute myocardial infarction (AMI) serum exposure elicits a distinct transcriptional signature in primary human macrophages. A) Human cardiac AMI tissue stained for von Willebrand Factor (brown) and haematoxilin (blue). 10× magnification of the healthy and ischemic region within the same heart. Scalebar is 100 µm. B) Schematic study design. C) Differentially expressed genes (DEGs) in AMI‐mac versus Ctrl‐mac (*n* = 47 AMI, *n* = 20 ctrl). D) Transcriptome‐based principal component analysis (PCA) plot of AMI‐mac and Ctrl‐mac. E) Area under the receiver operating characteristics curve (ROC AUC) and accuracy (Mean ± SD) in support vector machine (SVM), random forest (RF), and XGBoost (XGB) classification (*n* = 47 AMI, *n* = 20 ctrl) using transcriptomics data. F) Differential expression levels of 36 common feature selected genes in AMI‐mac versus Ctrl‐mac.

AMI causes significant vascular leakage in the heart, which we confirmed by visualizing the extravascular presence of von Willebrand Factor within the AMI region of the heart (**Figure** **1**A). We set out to investigate how macrophages, highly sensitive to environmental cues, would be affected upon direct contact with these blood components. Primary CD14^+^ monocyte‐derived macrophages from at least six different healthy volunteers were pooled to account for interdonor variability. Macrophages were verified to be bona fide M0 macrophages which could be polarized with interferon *γ* (IFNγ) or interleukin 4 (IL‐4) to express signature M1 and M2 markers (Figure S1, Supporting Information). We first tested a series of serum dilutions in a small‐scale phagocytosis assay. At 20% dilution, serum incubation was found to be optimal for in vitro experiments, as it was not causing reduction in cell counts while giving a maximal signal (Figure S2A,B, Supporting Information). Duration of serum incubation had little effect on nuclear counts or sample variability (Figure S2C,D, Supporting Information). Therefore, we chose to incubate macrophages for 24 h to optimally mimic the imprinting occurring during the initial phase of post‐AMI inflammation and repair. A discovery cohort of 50 AMI patient sera was built, selected from the POSTEMI study, a large and extensively characterized ST‐elevation myocardial infarct patient cohort with detailed clinical 4 months follow‐up;^[^ ^7^ ^]^ sera from 20 risk factor matched healthy volunteers served as control (**Table** **1**). All AMI patient sera were obtained within a couple of hours after symptom onset (median 2.8 h) and showed variable expression of signature cytokines (Figure S3, Supporting Information). Next, human primary monocyte‐derived macrophages from healthy volunteers were exposed to 1:5 diluted serum from AMI or control patients and extensively phenotyped (Figure 1B).

**Table 1:** Characteristics of discovery cohort (control and acute myocardial infarction (AMI) serum samples)

	Control	AMI (POSTEMI)	*p*‐value
Number of patients	20	50	n.a.
Age [years]	58.0 ± 1.4	59.0 ± 1.5	0.67^a)^
BMI^*^ [kg cm^−2^]	26.1 ± 0.9	27.3 ± 0.4	0.15^a)^
Male/female [%]	10/10 [50%]	44/6 [88%]	0.0013^b)^
Total plasma cholesterol [mmol L^−1^]	5.5 ± 0.1	5.1 ± 0.2	0.08^a)^
Smoking (*y*/*n*, [%])	9/11 [45%]	23/27 [46%]	0.9^b)^

^*^ Body mass index. Data is presented as mean ± SEM. *p*‐values were calculated with standard Student's *t*‐test (^a)^) or Chi‐square test (^b)^).

We deployed an integrated transcriptional and functional profiling strategy to provide a highly granular and unbiased view on macrophage phenotypic changes (Figure 1B). Serum‐exposed macrophages displayed high expression of prime macrophage signature genes (e.g., *Cd11b, Cd36, Cd44, Mrc1, MafB, Trem2, Ssp1, Marco*, and *Cd163)*, while dendritic cell (e.g., *Cd1a, Cd1c, Cd80*, and *Zbtb46*)) or monocyte markers (*Sell, S100A9, S100A8, Fcar, SerpinB2*, and *Tnfaip3)* were barely or un‐detectable. Initial data exploration immediately revealed transcriptional differences between AMI serum exposed and control serum exposed macrophages (AMI‐ and Ctrl‐mac; Figure 1C). Principal component analysis (PCA) confirmed this notion (Figure 1D). In further support, three well‐known machine learning classifiers support vector machine (SVM), random forest (RF), and XGBoost (XGB) efficiently segregated the study arms based on mRNA expression, with classification accuracies and areas under the curve (AUC) of close to 95% under cross‐validation (Figure 1E). Subsequent feature selection identified a 36‐membered gene signature for AMI‐induced reprogramming of macrophages (Figure 1F, **Table** **2**). Interestingly, AMI serum exposed macrophages (AMI‐mac) overexpressed *Srpx* and *Mfge8*, both involved in phagocytosis and wound healing.^[^ ^8^ ^]^ Moreover, the AMI signature was enriched in proteasome complex proteins, whereas DNA‐repair and cell cycling genes *Brip1*, *Plk4*, and *Ddias*, as well as cell–cell contact modulators *Cdh1* and *Adam12* were downregulated. Overall, this signature indicated that macrophages adapt their phagocytic, metabolic and cell cycling programs following AMI serum exposure.

**Table 2:** A total of 36 common feature‐selected genes characterizing acute myocardial infarction (AMI) induced reprogramming

Gene symbol	Log2 fold change ctrl‐AMI	Adjusted *p*‐value	Full name
SRPX	1.59	2.5 × 10^−22^	Sushi repeat‐containing protein
MFGE8	1.39	1.8 × 10^−17^	Lactadherin
GLRX	0.62	8.5 × 10^−18^	Glutaredoxin‐1
NQO1	0.52	2.5 × 10^−15^	NAD(P)H dehydrogenase [quinone] 1
CYB5R1	0.46	1.4 × 10^−15^	NADH‐cytochrome b5 reductase 1
P4HA2	0.45	2.5 × 10^−22^	Prolyl 4‐hydroxylase subunit Alpha‐2
PILRA	0.43	1.2 × 10^−16^	Paired immunoglobulin like Type 2 receptor alpha
PSMB4	0.43	8.4 × 10^−17^	Proteasome 20S subunit Beta 4
S100A10	0.41	1.2 × 10^−14^	S100 calcium binding Protein A10
NUCB1	0.39	2.5 × 10^−15^	Nucleobindin‐1
PSMB7	0.37	5.1 × 10^−14^	Proteasome 20S subunit Beta 7
PSMB1	0.37	1.0 × 10^−18^	Proteasome 20S subunit Beta 1
FTH1	0.37	7.7 × 10^−16^	Ferritin heavy chain
TDP2	0.37	3.9 × 10^−19^	Tyrosyl‐DNA phosphodiesterase 2
NBDY	0.34	8.0 × 10^−12^	Negative regulator of P‐body association
RAC1	0.33	6.2 × 10^−15^	Rac family small GTPase 1
TSPAN31	0.31	3.1 × 10^−12^	Tetraspanin 31
TCTA	0.31	5.5 × 10^−15^	T‐cell leukemia translocation altered
VMP1	0.28	8.4 × 10^−17^	Vacuole membrane Protein 1
PSMD1	0.27	8.6 × 10^−17^	Proteasome 26S subunit, non‐ATPase 1
TMEM50A	0.27	2.5 × 10^−16^	Transmembrane Protein 50A
SDCBP	0.26	3.3 × 10^−17^	Syntenin‐1
PSMD2	0.25	1.2 × 10^−13^	Proteasome 26S subunit, non‐ATPase 2
TAF7	0.25	6.5 × 10^−17^	TATA‐Box binding protein associated Factor 7
VCP	0.19	3.9 × 10^−13^	Transitional endoplasmic reticulum ATPase
SUPT16H	−0.20	6.0 × 10^−12^	SPT16 homolog, facilitates chromatin remodeling subunit
RCC1	−0.38	9.5 × 10^−12^	Regulator of chromosome Condensation 1
CAD	−0.46	1.8 × 10^−17^	Carbamoyl‐phosphate synthetase 2, aspartate transcarbamylase, and dihydroorotase
PFAS	−0.49	4.2 × 10^−17^	Phosphoribosylformylglycinamidine synthase
NCAPD2	−0.64	3.9 × 10^−16^	Non‐SMC Condensin I complex subunit D2
ANKS6	−0.85	4.0 × 10^−14^	Ankyrin repeat and sterile alpha motif domain containing 6
DDIAS	‐0.93	2.4 × 10^−13^	DNA damage‐induced apoptosis suppressor protein
PLK4	‐1.13	5.5 × 10^−12^	Serine/threonine‐protein kinase PLK4
ADAM12	‐1.15	2.9 × 10^−14^	A Disintegrin and metalloproteinase domain‐containing protein 12
BRIP1	‐1.38	1.6 × 10^−11^	Fanconi anemia group J protein
CDH1	‐1.45	6.9 × 10^−13^	Cadherin‐1

**Figure 2:**
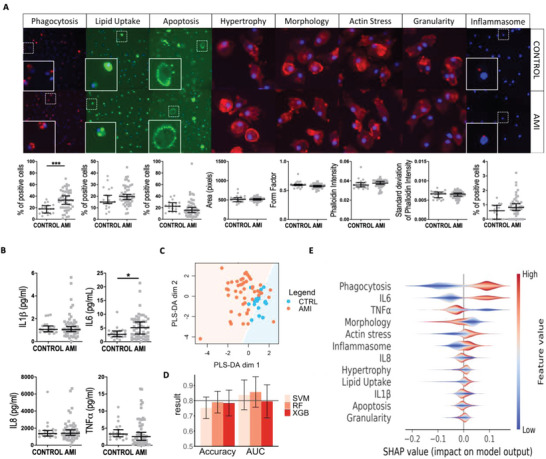
Acute myocardial infarction (AMI) and control serum exposed macrophages are functionally different. A) High content screening images with image cytometry data graphs for phagocytosis, lipid uptake, apoptosis, hypertrophy^#^, morphology^#^, actin stress^#^, granularity^#^, and inflammasome^##^ assays in AMI‐mac versus Ctrl‐mac in 10×, ^#^40×, or ^##^20× magnification. Dotted areas are enlarged in inserts. *n* = 50 AMI, *n* = 20 ctrl. Scalebar is 20 µm. B) Macrophage cytokine secretion after 24 h AMI or control serum exposure. *n* = 50 AMI, *n* = 20 ctrl. C) Partial least squares discriminant analysis (PLS‐DA) based on Macroscreen data. D) Area under the receiver operating characteristics curve (ROC AUC) and accuracy (Mean ± SD) in support vector machine (SVM), random forest (RF), and XGBoost (XGB) classification using Macroscreen data. E) SHapley Additive Explanations (SHAP) calculated feature importances of Macroscreen functionalities to the classification model RF. *p < 0.05, **p < 0.01, ***p < 0.001.

Next, we deployed the Macroscreen, an integrated high content functional mapping platform to establish phenotypic consequences associated with AMI serum exposure. AMI‐mac showed significantly increased phagocytic capacity and interleukin 6 (IL‐6) secretion and, to a lesser extent enhanced lipid uptake and inflammasome activity (**Figure** **2**A,B). Partial least squares discriminant analysis (PLS‐DA) as well as three Macroscreen data‐based machine learning classifiers revealed a clear separation of AMI‐mac and Ctrl‐mac functional profiles (Figure 2C,D). Men are predisposed to develop cardiac disease, we therefore ensured gender was not a confounder in our analysis, as demonstrated by gender‐colorcoding our sample distribution plots (Figure S4, Supporting Information). Phagocytic capacity and IL‐6 secretion contributed most to the AMI‐induced phenotype (Figure 2E). Thus, both the functional profiling and transcriptional data pointed to increased phagocytosis and immune activation by AMI serum exposure. Validation of the functional reprogramming in a second, independent human AMI versus healthy control serum cohort (see **Table** **3**, *n* = 47 AMI, *n* = 25 control) essentially confirmed AMI‐induced functional changes, except for macrophage actin stress fiber density and granularity (Figure S5A–C, Supporting Information). In support, applying the discovery cohort based classification model on the functional data of the validation cohort resulted in comparable AUC and accuracy values of at least 0.7 and 70%, respectively (Figure S5D, Supporting Information).

**Table 3:** Overview of human cohorts

Cohort name	Material	Data	Remarks	Computational analyses
Discovery cohort	50 AMI (>POSTEMI) and 20 control sera exposed human primary macrophages	RNA sequencing^a)^	3 AMI samples had to be excluded due to low RNA concentration	DEGs PCA Classification modeling Feature selection WGCNA GO network
		Macroscreen functional profiling^a)^	All AMI and ctrl samples included	PLS‐DA Classification modeling SHAP feature impact
POSTEMI Small–Large			RNA sequencing data from the discovery cohort AMI samples only	DEGs WGCNA GOEA Gene regulatory network (GRN) Drug repurposing
Validation cohort	47 AMI and 25 control sera exposed human primary macrophages	Macroscreen functional profiling^a)^		PLS‐DA Validation of discovery cohort classification models
GSE59867	Circulating peripheral blood mononuclear cells (PBMCs) from 111 patients with AMI 6 h after the AMI and 46 control	Microarray^b)^		Validation of discovery cohort AMI vs. control differentially expressed module genes
GSE59867 HF/non‐HF subset	Circulating peripheral blood mononuclear cells (PBMCs) of a subset of the 111 GSE59867 AMI patients 6 h after the AMI, containing 9 future heart failure (HF) and 8 future non‐heart failure (non‐HF) patients			Validation of discovery cohort POSTEMI‐Large vs. POSTEMI‐Small differentially expressed module genes

^a)^ Datasets generated within this study.

^b)^ Publicly available dataset.

### 2.2 Macrophage Exposure to AMI Serum Induces Immune, Mitogenic, and Metabolic Gene Programs

**Figure 3:**
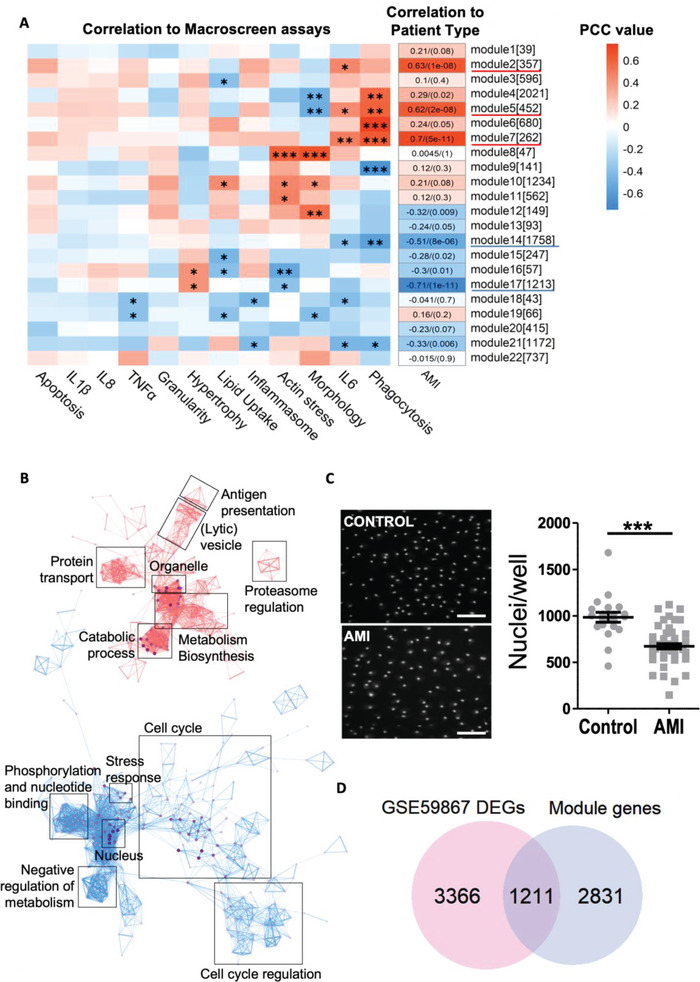
Acute myocardial infarction (AMI) serum exposure induces immune activation and metabolic programs, while cell cycling is suppressed. A) Pearson correlation coefficients (PCC) between AMI‐mac and Ctrl‐mac weighted gene co‐expression network analysis (WGCNA) modules [module size] and Macroscreen functionalities. *p*‐adj.: * < 0.05, ** < 0.01, *** < 0.001. PCC numbers (*p*‐adj.) are added for AMI. B) Gene ontology (GO) term interaction network of AMI‐correlating WGCNA modules M2, M5, M7, M14, and M17. C) Nuclear counts (mean ± SEM) and 10× high content screening images of Hoechst‐labeled nuclei of AMI‐mac and Ctrl‐mac. *n* = 50 AMI, *n* = 20 ctrl. ***p < 0.001. Scalebar is 50 µm. D) Significantly overlapping differentially expressed genes (DEGs) between AMI‐correlating WGCNA module genes and AMI‐associated DEGs in independent peripheral blood mononuclear cell (PBMC) mRNA dataset GSE59867 (hypergeometric testing; *p* = 1.62 × 10^−26^).

To identify coherent pathways driving the functional changes observed in AMI‐mac, weighted gene co‐expression network analysis (WGCNA) was carried out on the discovery cohort RNA sequencing dataset. Gene modules M5 (fatty acid oxidation, catabolic processes), M7 (antigen presentation, membrane organization), and M14 (cytoskeletal changes) correlated strongly with AMI serum exposure, as well as with macrophage phagocytosis capacity and IL‐6 secretion (**Figure** **3**A, Figure S6A, Supporting Information). Gene ontology (GO) enrichment analysis of AMI‐associated modules M2 (proteasome) and M17 (cell cycle regulation) suggested additional functional adaptation of macrophages to AMI serum exposure (Figure 3A, Figure S6A, Supporting Information). Again, we ensured our data did not suffer from a gender bias, as only M17 showed a weakly significant correlation with gender, which can only explain a small part of its observed AMI correlation (Table S1, Supporting Information). Combined, even despite the 1:5 dilution, macrophage exposure to AMI serum led to pronounced immune activation, metabolic changes, and suppression of cell cycle control (Figure 3B). Indeed, macrophage counts in culture, a surrogate measure of macrophage's mitogenic activity, were strongly reduced in AMI‐mac versus Ctrl‐mac (Figure 3C). Unexpectedly, transcriptomic data did not reveal an efferocytosis‐associated gene module specifically active in AMI‐mac, even though it is an important macrophage function during the initial AMI repair process. Efferocytosis actors such as TIM2, MerTK, or STAB1 were not differentially expressed upon AMI serum exposure. Moreover, uptake of apoptotic Jurkat T‐cells was unchanged in AMI‐ versus Ctrl‐mac (Figure S7, Supporting Information).

Next, we sought to verify the in vivo relevance of the observed AMI‐serum induced reprogramming, interrogating gene expression of peripheral blood mononuclear cells (PBMCs) from an AMI cohort sampled 6 h postinfarct (GSE59867).^[^ ^9^ ^]^ No significant overlap was found between the total of differentially expressed genes (DEGs) in our dataset and GSE59867 6 h AMI‐ctrl DEGs. However, upon constricting our dataset to DEGs belonging to AMI‐associating modules, they showed 30% overlap (*p* = 1.62 × 10^−26^) with DEGs in PBMCs from ST‐elevation myocardial infarction (STEMI) patients (*n* = 111) versus control PBMC samples (*n* = 46) (Figure 3D).

### 2.3 AMI Serum Reprogramming of Macrophages Associates with Clinical Progression

**Figure 4:**
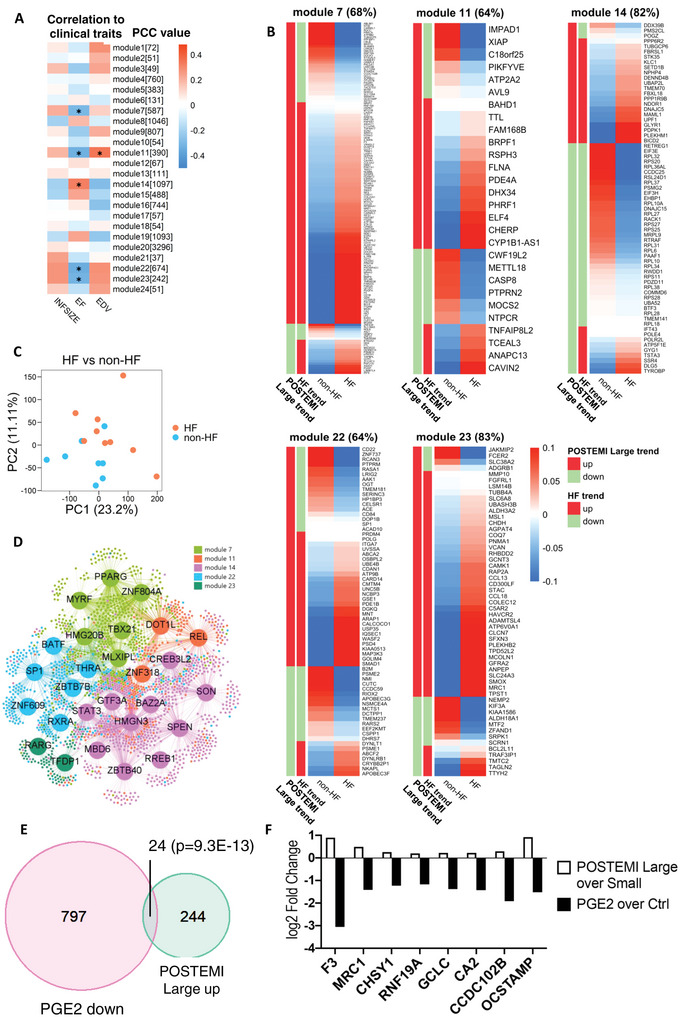
Acute myocardial infarction (AMI) serum reprogramming of macrophages associates with clinical progression. (A) Pearson correlation coefficient (PCCs) between AMI‐specific weighted gene co‐expression network analysis (WGCNA) modules [module size] and infarct size (INFSIZE), ejection fraction (EF), and end‐diastolic volume (EDV). *p < 0.05. B) Expression heatmap of AMI‐specific WGCNA module genes in GSE59867 (AMI patients with versus without subsequent heart failure, HF). Similar up‐ (red sidebar) or down‐ (green sidebar) regulation between the two datasets is indicated. C) Principal component analysis (PCA) plot of gene expression data in GSE59867 (HF versus non‐HF peripheral blood mononuclear cells (PBMCs)). D) Regulatory network showing the top 1% drivers of WGCNA modules (color‐coded) that correlated with cardiac performance 4 months after AMI. E) Overlap between prostaglandin E2 (PGE2) downregulated and POSTEMI‐Large upregulated genes (hypergeometric testing; *p* = 9.36 × 10^−13^). F) Log2 fold changes for the POSTEMI‐Large top‐ranked differentially expressed genes (DEGs; *p* < 0.001).

We next assessed whether the AMI‐induced signature correlated with patient prognosis (cardiac function 4 months post‐AMI). The AMI arm of the discovery cohort was designed to contain 25 patients with a small infarct size (POSTEMI‐Small, *n* = 25), while the other 25 patients suffered larger infarcts, with left ventricular reduced ejection fractions (EF) and higher end‐diastolic volumes (EDV), associated with post‐MI ventricular dysfunction (POSTEMI‐Large, *n* = 25, **Table** **4**).^[^ ^7^ ^]^ WGCNA of the AMI‐only dataset revealed four adverse (M7: endocytosis, M11: type I interferon response, M22: no GO terms associated, and M23: metal ion responses) and one protecting modules (M14: stress fiber and granule formation) to be significantly associated with EF and EDV (**Figure** **4**A, Figure S6B, Supporting Information). We used a subset of the GSE59867 PBMC cohort, consisting of patients with low and high left ventricular EF and termed non‐HF and HF (heart failure) respectively by the authors, to validate these findings. Interestingly, differential expression between large and small infarct genes from our “ventricular dysfunction” associating modules was significantly mirrored (64–83%) by baseline PBMCs (6 h after infarct) from these HF versus non‐HF patients (Figure 4B).^[^ ^9^ ^]^ This underpins the in vivo relevance of our ex vivo transcriptional imprint of serum on monocytes for effective cardiac repair. Indeed, the GSE59867 data showed a clear segregation of non‐HF versus HF patients (Figure 4C). Moreover, the recently published single cell spatial transcriptomics dataset allowed us to validate our AMI‐serum induced gene programs in myeloid cell subsets in human infarcted hearts (Figure S8, Supporting Information).^[^ ^10^ ^]^ We observed a strong enrichment of our POSTEMI‐Small signature in the healthier zones (Control, Remote) of the infarcted heart (Figure S8A, Supporting Information). In addition, POSTEMI‐Small and Large signatures were preferentially enriched in different cardiac macrophage subsets: LYVE^+^FOLR^+^ versus CCL18^+^ macrophages respectively (Figure S8B,C, Supporting Information). While these newly discovered subsets are not yet well described, our observations reveal a strong spatial and subset‐specific enrichment of AMI‐serum induced macrophage genes.

**Table 4:** Characteristics of POSTEMI acute myocardial infarction (AMI) patients with small versus large infarct

	POSTEMI‐Small	POSTEMI‐Large	*p*‐value
Age [years)	58.6 ± 2.3	59.5 ± 1.9	0.76^i)^
Male/Female [%]	20/5 [80%]	24/1 [96%]	0.09^ii)^
BMI [kg cm^−2^]	27.7 ± 0.7	27.0 ± 0.6	0.43^i)^
Total plasma cholesterol [mmol L^−1^]	5.1 ± 0.3	5.1 ± 0.2	0.95^i)^
Smoking (*y*/*n*, [%])	12/13 [48%]	11/14 [44%]	0.79^ii)^
Hypertension (*y*/*n*, [%])	5/20 [20%]	5/20 [20%]	0.99^ii)^
Diabetes (*y*/*n*, [%])	4/21 [16%]	1/24 [4%]	0.17^ii)^
Infarct size	2.0 ± 0.3	40.8 ± 1.9	< 0.0001^i)^
Left ventricular ejection fraction [%]	64.8 ± 1.0	36.1 ± 2.1	< 0.0001^i)^
Left ventricular end‐diastolic volume [mL]	161.0 ± 30.4	248 ± 12.2	< 0.0001^i)^

Data is presented as mean ± SEM. *p*‐values were calculated with standard Student's *t*‐test (^i)^) or Chi‐square test (^ii)^). Infarct size, left ventricular ejection fraction (EF), and end‐diastolic volume (EDV) were measured by cardiac magnetic resonance (CMR) 4 months after event time.

Based on gene expression data from AMI‐mac and Ctrl‐mac we constructed a regulatory network and identified 27 regulators of the significantly POSTEMI‐Large correlating modules (i.e., M7, M11, M14, M22, and M23) (Figure 4D). POSTEMI‐Large associated gene programs implicated retinoic acid receptor *α* and *γ* (RXR*α*, RAR*γ*) signaling, concordant with their reported role in adverse cardiac remodeling.^[^ ^11^ ^]^ Of note, RAR binding partner peroxisome proliferator‐activated receptor *γ* (PPAR*γ*) and c‐Rel were also identified as key drivers, pointing to alternative macrophage activation in AMI patients with poor prognosis.^[^ ^12^ ^]^ Identifying the key regulators of the AMI‐serum induced imprinting offers unique opportunities for therapeutic intervention.

The LINCS1000 cell perturbation database contains expression profiles of cells exposed to thousands of compounds and therapeutic agents.^[^ ^13^ ^]^ Within this repository, THP‐1 human monocyte‐derived cells most closely relate to the primary macrophages used in our study. Applying in silico drug screening we selected leads able to neutralize detrimental gene programs induced by the AMI circulatory environment. Top candidates with cytostatic or broad‐spectrum activity profiles, such as several PI3kinase family inhibitors (**Table** **5**), have low clinical potential for post‐AMI treatment. The transcriptional signatures of SCH79797, a thrombin receptor proteinase activated receptor 1 (PAR1) antagonist, and of PGE2 receptor agonist 16,16‐dimethylprostaglandin‐e2 in THP‐1 macrophages also mirrored the “POSTEMI‐Large” phenotype efficiently (connectivity score of −0,85 and −0.77, respectively, Table 5). To confirm, we incubated human primary macrophages for 24 h with PGE2, an analogue of 16,16‐dimethylprostaglandin‐e2, and analyzed macrophages expression profiles by RNAsequencing. PGE2‐downregulated and POSTEMI‐Large upregulated genes showed significant overlap (Figure 4E); with differential gene expression of POSTEMI‐Large versus Small pointing to osteoclastogenesis, a process suppressed by PGE2 (Figure 4F).^[^ ^14^ ^]^

**Table 5:** Drug repurposing screen results

Drug name	Cell type	Dose [m]	Duration [h]	Connectivity score
Wortmannin	THP1	10 × 10^−6^	6	−1
ABT‐751	THP1	10 × 10^−6^	6	−0.92
TUL_XIX002	THP1	10 × 10^−9^	6	−0.88
Phorbol‐12‐myristate‐13‐acetate	THP1	10 × 10^−6^	6	−0.87
SCH‐79797	THP1	10 × 10^−6^	6	−0.85
MW‐STK33‐100	THP1	1 × 10^−9^	24	−0.80
Sparfosic‐acid	THP1	80 × 10^−6^	6	−0.79
MW‐STK33‐97	THP1	10 × 10^−6^	24	−0.78
BRD‐K95985487	THP1	10 × 10^−6^	24	−0.78
BRD‐K49010888	THP1	1 × 10^−6^	24	−0.78
16,16‐dimethylprostaglandin‐e2	THP1	10 × 10^−6^	6	−0.77
BRD‐K95985487	THP1	3 × 10^−6^	6	−0.73
MW‐STK33‐2B	THP1	100 × 10^−9^	6	−0.73
Simvastatin	THP1	10 × 10^−6^	6	−0.71
MW‐STK33‐1C	THP1	10 × 10^−6^	24	−0.69
Wortmannin	THP1	10 × 10^−6^	24	−0.68
KU‐0063794	THP1	10 × 10^−6^	6	−0.68
BRD‐K95985487	THP1	10 × 10^−6^	6	−0.68
BRD‐K76188144	THP1	100 × 10^−6^	6	−0.67
BRD‐K95985487	THP1	3 × 10^−6^	24	−0.67
TUL_XIX002	THP1	100 × 10^−9^	6	−0.66
BG‐STK33‐03	THP1	100 × 10 ^−9^	6	−0.66
Gemcitabine	THP1	100 × 10^−9^	6	−0.66
BG‐STK33‐03	THP1	1 × 10^−9^	24	−0.65
L‐690488	THP1	10 × 10^−6^	6	−0.65
Methyl‐fasudil	THP1	10 × 10^−6^	6	−0.64
BRD‐K50776152	THP1	40 × 10^−6^	6	−0.63
PX‐12	THP1	30 × 10^−6^	6	−0.63
CD‐437	THP1	10 × 10^−6^	6	−0.63
Troglitazone	THP1	10 × 10^−6^	6	−0.63
TUL_XIX003	THP1	1 × 10^−9^	24	−0.63
TUL_XIX003	THP1	10 × 10^−6^	6	−0.62
BRD‐K05402890	THP1	500 × 10^−9^	6	−0.62
Wortmannin	THP1	10 × 10^−6^	6	−0.61
TUL_XIX002	THP1	1 × 10^−9^	6	−0.61
NSC‐95397	THP1	10 × 10^−6^	6	−0.61
MW‐STK33‐100	THP1	10 × 10^−9^	6	−0.61
Valdecoxib	THP1	100 × 10^−6^	6	−0.60

Candidates with a connectivity score <−0.60 from the LINCS THP1 drug repurposing screen are included.

## 3 Discussion

Macrophages are firmly established as key orchestrators of cardiovascular diseases. As macrophages are plastic cells notorious for their adaptation to environmental context changes, we investigated whether the systemic environment of acute cardiac ischemia would rewire macrophages in a disease‐relevant manner. Integrating high content analysis based functional profiling and transcriptomics analysis, we here demonstrate that exposure of macrophages to the blood of subjects having suffered a myocardial infarct leads to extensive reprogramming toward a trauma healing phenotype with potential repercussions on disease prognosis.

AMI is associated with marked changes in serum protein composition, including IL‐6, B‐type natriuretic peptide (BNP), chemokine (C motif) ligand (XCL1), and NF‐kappa‐B essential modulator (NEMO).^[^ ^1b^ ^]^ In addition, ischemia‐associated release occurs of many known inflammatory mediators such as heat shock protein‐70^[^ ^15^ ^]^ and 60^[^ ^16^ ^]^ and high mobility group box‐1.^[^ ^17^ ^]^ Several of these factors have been proposed as biomarkers of cardiac injury.^[^ ^1b^ ^]^ AMI‐trauma results in obvious vascular leakage, as we have here confirmed again by demonstrating the extravascular presence of von Willebrand Factor in AMI cardiac tissue. Conceptually, we hypothesized that a fivefold serum dilution would emulate the diffusion‐limited exposure of macrophages in the cardiac inflamed tissue to blood components. We chose to work with primary human monocyte‐derived macrophages to evaluate effects of the AMI systemic inflammatory environment on macrophages. We argued this approach would resemble most closely the in vivo situation. Moreover, working with accessible monocyte‐derived cells makes intervention in their potentially detrimental AMI programming an attractive option. As we show here, the AMI‐associated systemic, proinflammatory environment can indeed impact macrophage transcriptional and functional make‐up and thus their capacity to respond to the cardiac trauma. These effects were observed with fivefold dilution of the AMI serum, suggesting that even interstitial macrophages deeper in tissue, especially under inflammatory conditions, will undergo rewiring.

At the transcriptional level, AMI serum exposure affected significant changes pointing to increased proteasome activity, cell cycle regulation, and immune activation. The induction of proteasome and suppression of cell cycle gene programs as well as significant metabolic changes in AMI‐sera exposed macrophages, were supportive of the reported debris‐clearing, immune activating role of macrophages in the infarcted heart.^[^ ^18^ ^]^ Confirming the immune activation, we observed consistent increases in macrophage IL‐6 secretion after 24 h exposure to AMI serum. Of note, IL‐6 was also increased directly in the AMI serum compared to controls in the POSTEMI cohort. In accordance with our data, we interrogated a recently published AMI serum proteomics open access dataset by gene ontology overrepresentation analysis and found IL‐6 production to be one of the key terms most strongly associating with the AMI serum specific proteome.^[^ ^19^ ^]^

To comprehensively map environment‐induced changes in macrophage functionality, we employed high content screening (HCS). While HCS has been used successfully for drug or RNA interference screening purposes,^[^ ^20^ ^]^ applications generally rely on assessment of a single functional parameter. The applied multiparameter screening strategy, especially when integrated with transcriptional analysis, provides a much more granular and unbiased insight into the macrophage's phenotypic changes. Deploying advanced machine learning approaches, we identified key features of AMI‐serum challenged human macrophages for two independent AMI patient cohorts: increased phagocytosis and inflammation (IL‐6 and TNF*α*), resonating the AMI serum associated transcriptional changes. Inhibition of IL‐6 and TNF*α* to improve cardiac healing has yielded variable results in clinical trials.^[^ ^21^ ^]^ Thus, IL‐6 and TNF*α* production in AMI serum exposed macrophages should be interpreted within the full framework of AMI‐induced reprogramming, offering new options for therapeutic design. Slightly surprisingly, efferocytosis turned out to be unaffected by AMI serum exposure. Efferocytosis is however an extremely conserved feature of macrophages,^[^ ^22^ ^]^ and we postulate here that its machinery is fully able to effectively process apoptotic cells in the early phase of AMI, regardless of modulating factors contained in AMI or control serum. Conceivably, the observed phenotypic changes upon AMI serum exposure mirror effects of cardiac ischemia on cardiac macrophages in situ, as blood‐born and heart‐resident macrophages in the inflamed edematous infarct zone will both be exposed to plasma components. Of note, functional changes of serum exposure could be corroborated with plasma from the same AMI patient cohort, excluding those changes are attributable to coagulation. Inflammatory monocytes are a main source of monocyte‐derived macrophages accumulating at the infarct site, as shown repeatedly.^[^ ^2^, ^23^ ^]^ The fact that we could partly validate this AMI signature in a cohort of PBMCs taken 6 h after infarct pleads for this notion. Thus, the AMI‐mac signature is mirroring changes seen in circulating PBMCs in AMI patients, especially considering the heterogenous nature of this cell preparation. Nevertheless, it remains to be firmly established whether (resident and monocyte‐derived) cardiac macrophages will undergo similar reprogramming in AMI in situ.

In addition to characterizing the response of macrophages upon contact with the AMI systemic environment, we explored differences between patients having suffered larger infarcts associated with significant post‐MI ventricular dysfunction (POSTEMI‐Large) and patients having suffered smaller infarcts (POSTEMI‐Small). Five AMI sera‐exposed macrophage gene modules correlated with clinical features such as left ventricular EF. Interestingly, genes from those modules showed significant overlap with DEGs in PBMCs (6 h after infarct) from patients with larger versus smaller infarcts in the GSE59867 cohort. In addition, we have found enrichment of our POSTEMI‐Large and Small gene signatures in specific populations of cardiac macrophages in human infarcted hearts. This data suggests that context‐induced gene programs, operational in monocytes and macrophages immediately after AMI, could influence patient prognosis.

Next to implications of AMI associated macrophage imprinting for infarct healing, myocardial infarction (MI) is also known to predispose to cardiovascular events in the immediate aftermath, by inducing plaque growth, destabilization,^[^ ^2a^ ^]^ and haemorrhage.^[^ ^24^ ^]^ While this may be partly ascribed to post‐AMI monocytosis, the prevalent procoagulant state, and hemodynamic factors, our results also suggest a role for AMI‐induced reprogramming of blood‐born or plaque‐resident macrophages, rendering them more phagocytic, immune‐activated, and metabolically challenged.

Indeed, macrophage subsets enriched in plaque of symptomatic carotid endarterectomy patients shared characteristics with our AMI‐induced “unfavourable” phenotype, i.e., enhanced phagocytosis and PPAR‐dependent alternative macrophage activation.^[^ ^25^ ^]^ This led us to speculate that the AMI‐associated macrophage imprint may resonate with plaque inflammation. The notion that organ‐resident macrophages adapt to systemic environmental changes with potential impact on future health increasingly gains momentum,^[^ ^26^ ^]^ but in a human CVD setting, evidence has so far been lacking. Regrettably, scRNA‐seq or bulk gene expression studies on isolated human plaque macrophages from infarct patients are not yet available, hampering direct assessment of our ex vivo AMI signature in plaque macrophages. Nevertheless, context imprinting in AMI trauma response, as here described, offers a unique opportunity for therapeutic steering of macrophage responses toward a more favorable direction. Our network‐guided in silico drug screening method identified PGE2 receptor agonist 16,16‐dimethylprostaglandin‐e2 as a possible drug candidate. Indeed, prostaglandin E_2_ receptor (EP 2) signalling in macrophages is critical for cardiac repair and functional recovery in infarcted mice,^[^ ^27^ ^]^ underpinning the therapeutic potential for short‐term PGE2 treatment after AMI. Our findings warrant future studies on PGE2's potential to reverse detrimental reprogramming of monocyte‐derived macrophages by AMI.

Importantly, our study is limited by its rather small cohort sizes, which do not represent the diversity of the whole population. Nevertheless, the robust replication of our findings in an independent patient cohort suggests that our results have general validity. As we aimed to study effects of the circulatory environment on cells and not vice versa, our patient cohorts were blood‐based, whereas our readout cells were derived from healthy donors. Future studies should ideally complement this work by investigating the AMI‐related imprinting in monocytes and macrophages isolated from patients and control individuals at different timepoints after AMI. Still, our work clearly identifies the systemic circulation as an undervalued actor in macrophage behavior in post‐AMI repair.

In conclusion, our study is the first to demonstrate the transcriptional and functional rewiring of macrophages upon exposure to the AMI circulatory environment, to improved debris clearance and healing responses. Moreover, we identified gene programs and their regulatory circuitry, associating with poor patient prognosis, which could be targeted by PGE2 receptor agonists, offering an entirely new therapeutic approach for prevention of adverse postinfarct remodeling.

## 4 Experimental Section

### Study Design

Human primary monocyte‐derived macrophages from healthy volunteers were exposed for 24 h to a 1:5 dilution of human patient serum, and changes in transcription and function were analyzed. Patient sera (discovery cohort) were obtained either from control patients or patients suffering an AMI. Four months after initial AMI event, patients with regained and worsened cardiac function showing signs of heart failure were scored to allow linkage of the AMI‐serum induced macrophage effects to clinical prognosis. Sera‐exposed macrophages were analyzed by RNA sequencing and a multiparameter functional screening method based on both high content imaging and multiplex ELISA (Macroscreen). Functional screening data was validated using an independent patient cohort (Validation cohort). The RNA sequencing dataset was computationally analyzed and integrated with functional data and patient characteristics to identify meaningful gene programs relating to AMI serum exposure. Finally, a drug repurposing screen identified a potential therapeutic with the ability to reverse unfavorable clinical effects. An overview of all cohorts mentioned in this manuscript, as well as how they are used, is presented in Table 3.

### Human Cohort Design—Discovery Cohort

The discovery cohort consisted of serum from 50 AMI patients and 20 control healthy volunteers (Table 1). Fifty AMI patients with STEMI were selected from the POSTEMI (Postconditioning in STEMI) study to be included in the discovery cohort. The POSTEMI study was a prospective, randomized, single‐center, open‐label clinical trial investigating the cardioprotective strategy of ischemic postconditioning, yielding neutral study results.^[^ ^7^ ^]^ The trial was approved by the Regional Committee for Medical Research Ethics, South‐East Norway on July 30, 2008, and registered (NCT00922675). The study was conducted in accordance with the Declaration of Helsinki, and all patients gave written informed consent. More information on patient inclusion and exclusion criteria is provided in the Supporting Information. This work aimed to (1) compare serum from AMI patients with controls and (2) compare serum from patients with large MI with serum from patients with small MI. To enable the second comparison, we designed the group of 50 AMI patients to contain 25 randomly selected patients from the highest quartile of MI and 25 patients from the lowest quartile, according to infarct size measured by cardiac magnetic resonance imaging (CMR, late gadolinium enhancement images) at 4 months follow‐up (POSTEMI‐Large and POSTEMI‐Small respectively, see Table 4). Median infarct size in % of left ventricular volume (measured by CMR) was 2.1% and 38.3%, respectively. Left ventricular EF was calculated by assessment of the volumes of the endocardial contours in diastole and systole of the short axis images, and EDV by epicardial tracing during diastole, as described previously in detail.^[^ ^7^ ^]^ For reference, this work used 20 control serum samples, collected from healthy volunteers (healthcare workers, healthy according to disease history) and matched for age, BMI, smoking status, and total plasma cholesterol levels at admission were added to these 50 POSTEMI patient samples to obtain the full discovery cohort (Table 1). Cardiovascular disease history, elevated hsCRP (>10 mg mL^−1^) and the presence of carotid atherosclerosis (assessed by ultrasound) were considered exclusion criteria. Inclusion of control samples was approved by the Oslo University Hospital local ethical committee (ethical approval number 2017/2202). Serum samples from this discovery cohort were used to stimulate human primary macrophages which were analyzed by RNA sequencing and Macroscreen functional assays (Table 3).

### Human Cohort Design—Validation Cohort

The validation cohort consisted of serum from 47 AMI patients and 25 control healthy volunteers. Forty‐seven AMI patients were included in the validation cohort: 24 patients with non‐ST elevated MI (NSTEMI) and 23 patients with STEMI. AMI diagnosis was defined as a typical rise and fall of the cardiac biomarker troponin T with at least one value above the 99th percentile of the upper reference limit in patients presenting with symptoms of ischemia. STEMI patients adhered to the additional ECG criteria as described above. Control samples consisted of 25 age‐matched healthy volunteers (Mean age ± SEM: 63.9 ± 1.9 for AMI versus 62.5 ± 1.3 for controls). Cardiovascular disease history, elevated hsCRP (>10 mg mL^−1^) and the presence of carotid atherosclerosis (assessed by ultrasound) were considered exclusion criteria. Validation cohort buildup was approved by the Oslo University Hospital local ethical committee (ethical approval number S‐06172). Also for the validation cohort, serum samples were used to stimulate human primary macrophages, which were functionally profiled by the Macroscreen assay platform (See Table 3).

### Human Serum Sample Collection

In all subjects, blood was drawn at admission. In NSTEMI patients and controls, blood was collected from a peripheral vein; in STEMI patients, arterial blood was collected directly from the arterial cannula during cannulation of the arteria radialis or femoralis at the start of the acute coronary angiography and before PCI (median 2.8 h after symptom onset). Blood samples were collected into endotoxin‐free tubes without additives and centrifuged within 1 h after collection at 2500 g for 10 min after full coagulation at room temperature. Immediately following centrifugation, serum was collected and stored at −80 °C in multiple aliquots until analyses. Samples were thawed only once before use.

### Human Primary Monocyte‐Derived Macrophages Isolation and Culture

Buffy coats were collected from healthy volunteers of the Blood Bank (RWTH University Hospital Aachen, Germany). PBMCs were isolated using Ficoll–Paque gradient (Sigma). CD14^+^ monocytes were positively selected using CD14 MicroBeads (Miltenyi) according to manufacturer's protocol. Monocytes were pooled from 6 to 8 donors per assay, plated in Falcon 96‐well Black Imaging Microplates at a density of 75 000 cells per well in RPMI1640 (Thermofisher) supplemented with FCS (Gibco, 10%) and PenStrep (Gibco, 1%), and cultured in a controlled environment (37 °C, 5% CO_2_). Monocytes were differentiated to macrophages using rh‐MCSF (Immunotools, 100 ng mL^−1^) for 7 or 8 days with one medium change.

### Stimulation of Macrophages

Macrophages were stimulated by patient sera (denoted as AMI‐mac and Ctrl‐mac) and by PGE2, IL‐4 or Interferon gamma (IFN*γ*), followed by Macroscreen functional analysis and RNA sequencing. Details about stimulation, RNA isolation, and sequencing analyses can be found in Supporting Information.

### Macroscreen Functional Profiling of Stimulated Macrophages (High Content Assays)

Functional assays were performed using a high content analyzer BD Pathway 855 (BD Bioscience). Each 96‐well stimulated macrophage plate contained at least six control wells of nonstimulated macrophages, as well as technical control wells where fluorophores were omitted. All functional assays were performed at least in duplicate, exposing macrophages to serum samples from the complete discovery cohort (50 AMI patients and 20 control serum samples) or validation cohort (47 AMI patients and 25 control serum samples, Table 3). Image cytometry was performed on nine pictures per well at assay‐appropriate magnification (10×, 20×, or 40×), as indicated, and was analyzed with Attovision image analysis software (BD Bioscience) for apoptosis, phagocytosis, lipid uptake and inflammasome assays, and CellProfiler 3.1.8. (open source modular high‐throughput image analysis software^[^ ^28^ ^]^) for cell shape assessment. For all image analyses, Hoechst‐based nuclear identification served as input for primary object segmentation. Primary objects were enlarged using assay‐dependent analysis pipelines and fluorescence intensity was measured within each object. Attovision results were further processed using the DIVA software (BD Bioscience), enabling efficient gating of fluorophore‐positive cells. Threshold for positivity was determined by means of the technical control wells. Details on the methodology of functional and cytokine assays and Macroscreen statistics and preprocessing can be found in Supporting Information.

### RNA‐seq and Computational Analyses—RNA‐seq

RNA‐seq analysis was performed on the discovery cohort to evaluate the changes between AMI‐ or control sera‐stimulated macrophages at the gene level. Details about the RNA isolation, sequencing analysis, data preprocessing, quality control, as well as computational analyses including PCA and differential gene expression analysis (Tables S2 and S3, Supporting Information) can be found in the Supporting Information.

### Machine Learning Analyses—Feature Selection

Feature selection was performed on the complete discovery cohort RNA sequencing dataset using three methods. For minimal‐redundancy‐maximal‐relevance (mRMR^[^ ^29^ ^]^), 100 genes were sequentially selected using R package mRMRe (v2.1.0). With the maximal information coefficient‐based approach (MIC^[^ ^30^ ^]^), top 100 genes most associating with the AMI‐ctrl phenotype were calculated by the R package minerva (v1.5.8). Top 100 AMI‐mac versus Ctrl‐mac DEGs were identified based on the ranking of adjusted p‐values from low to high. Genes shared between the top 100 feature selected genes from mRMR, MIC and DEGs were intersected, resulting in a final list of 36 feature selected genes (Table 2).

### Machine Learning Analyses—Classification

PLS‐DA, a supervised linear classification model, provided in R package mixOmics (v6.8.1), was performed on Macroscreen functional data (discovery and validation cohort). Samples were plotted on the first two components. SVM (with platt scaling for probability prediction), RF, and XGB were applied on the RNA sequencing dataset of the discovery cohort and the Macroscreen functional data (for both discovery and validation cohort) using the scikit‐learn (v0.23.2) and xgboost (v1.3.0) packages (Python). A radial‐based function kernel was used in SVM. Other hyperparameters were set as default. Accuracy and area under the receiver operating characteristics curve (ROC AUC) were measured under stratified fivefold cross‐validation with 10 repeats.

### Machine Learning Analyses—Feature Importance

Feature importance was inferred by the SHapley Additive Explanations (SHAP) method, a game theoretic approach to explain the output of a model.^[^ ^31^ ^]^ SHAP values of each Macroscreen measurement were calculated by fitting an RF classifier on the training data followed by prediction on the testing samples (shap package v0.37.0, Python). The training/testing split was realized by stratified fivefold cross‐validation (10 repeats). Results from each run were aggregated to represent the feature contribution (Figure 2E).

### Network Analyses—Weighted Gene Co‐expression Network Analysis (WGCNA)

WGCNA (v1.68) was performed two times independently: 1) on the complete transcriptomics RNA sequencing dataset of the discovery cohort, including 47 AMI‐mac and 20 Ctrl‐mac (Table S4, Supporting Information, and Table 2) Within the same dataset, analyzing only the AMI‐mac transcriptomics data, including 23 POSTEMI‐Small and 24 POSTEMI‐Large exposed macrophages (Table S5, Supporting Information). Scale‐free property was achieved (scale‐free fitting index, i.e., the *R* ^2^ used in the function scaleFreeFitIndex in the WGCNA package: 0.91 and 0.89, soft threshold set to 8 and 6, respectively). Other parameters were set as default, resulting in 22 modules in the full dataset (module M22 contains genes that are not coexpressed), and 24 modules in the POSTEMI subcohort data (module M24 contains genes that are not coexpressed). For the full AMI/control dataset, Pearson correlation coefficients (with Student asymptotic *p*‐values) between the eigengene of each module and Macroscreen functional measurements as well as sample identity “AMI” was calculated. For the POSTEMI subcohort analysis, Pearson correlation coefficients (with Student asymptotic *p*‐values) between the eigengene of each module and CMR parameters infarct size, EF, and EDV were calculated.

### Network Analyses—Gene Ontology Enrichment Analysis (GOEA)

R package clusterProfiler (v3.12.0) was applied for enrichment analysis based on gene ontology resources. Enriched GO terms with log‐transformed *p*‐values of several highly correlating modules were selectively shown as bar plots. A complete list of the GO terms associating with WGCNA modules can be found in Table S6 (Supporting Information, full discovery cohort) and Table S7 (Supporting Information, subcohort POSTEMI Small–Large). *p*‐values were further corrected by the Benjamini and Hochberg procedure. A gene ontology network was created by BiNGO (v3.0.3), a plug‐in software provided in Cytoscape (v3.7.1). The FDR was set to 0.05 to filter out less significantly enriched GO terms. The Cytoscape plug‐in Enrichment Map (v3.2.0) was used to visualize the GO network. Modules correlating positively (M2, M5, and M7) and negatively (M14 and M17) with the AMI phenotype were separately analyzed and visualized by red and blue colors. Similarities between GO terms were calculated by the Jaccard Index and were further represented by weighted edges. The edge cut‐off was set to 0.25, and the node cut‐off *q*‐value was set to 0.1. GO clusters with similar biological functions were manually annotated.

### Network Analyses—Regulatory Network Construction

The gene regulatory network (GRN) was constructed using ARACNe (Java executable file from the Califano lab), an information theoretic‐based network inference framework.^[^ ^32^ ^]^ All 67 samples (47 AMI, 20 control) of the discovery cohort transcriptomics dataset were included. A list of 1639 (1018 contained in our data) transcription factors (TFs) retrieved from Lambert et al.^[^ ^33^ ^]^ was provided as candidate TFs, and all 12341 genes in our dataset were considered as targets. The ARACNe regulatory network was constructed using a bootstrapping strategy with *p*‐value as 1 × 10^−7^, data processing inequality (DPI) as 0.1, and resampling as 100. The GRN achieved scale‐free property, with a scale‐free fitting index of 0.839. To identify those regulators associated with adverse cardiac remodeling, a subnetwork was created by only preserving the members of POSTEMI Small–Large modules M7, M11, M14, M22, and M23 as regulating targets, thus containing 1018 candidate TFs and 2990 targets. Regulatory scores (RS) were defined as the total of all edge weights multiplied by its targets’ absolute log2 fold changes in expression between the POSTEMI‐Small and POSTEMI‐Large sample groups (see Table S8, Supporting Information). Based on RS ranking, the top 1% TF relative to each module's size was selected and visualized in the GRN using the Gephi software package (v0.9.2) with ForceAtlas 2 layout.

### Validation in Public Datasets

Differential expression of WGCNA module genes (full discovery cohort dataset) was validated in the GSE59867 dataset (microarray gene expression data from PBMCs isolated from 111 patients at different timepoints following ST‐elevated myocardial infarction and 46 control patients with stable coronary artery disease).^[^ ^9^ ^]^ DEGs between the 6 h time point after AMI and control samples were analyzed (absolute log2 fold change threshold > 0.15) and nonrandomness in gene overlap was evaluated using hypergeometric testing. For validation of the AMI‐specific WGCNA network, only the GSE59867 6 h PBMC samples from those AMI patients marked “heart failure” (HF, *n* = 9) and “non‐heart failure” (non‐HF, *n* = 8) were included. Briefly, 6 months after the initial myocardial infarction, these patients had the highest and lowest, respectively, level of plasma NT‐proBNP in combination with the lowest and highest, respectively, levels of LVEF (left ventricular EF). Differential expression levels of modules M7, M11, M14, M22, and M23, AMI‐specific WGCNA modules significantly correlating with clinical traits, were visualized between the HF and the non‐HF group. Within‐group expressions were averaged, and between‐group contrasts were highlighted by deducing the average expression in all patient groups.

Counts data from the human infarcted hearts scRNA‐seq dataset were retrieved from the Kuppe study data deposition.^[^ ^10^ ^]^ Only myeloid cells were selected for further analysis. The single‐sample GSEA (ssGSEA) was run to test the DEGs between POSTEMI‐Large versus POSTEMI‐Small in the heart scRNA‐seq dataset. For each cell and each DEG list, we obtained an enrichment score, and these values were further averaged per cell type to present the overall enrichment of a DEG list in the corresponding cell type. The ssGSEA was executed using the R package GSVA (v1.42.0).^[^ ^34^ ^]^

### Drug Repurposing Screen

The THP1 cell perturbation database from LINCS L1000^[^ ^13^ ^]^ was used for network‐guided drug screening. The original LINCS L1000 database contains >1 million mRNA expression signatures for 350 cell lines and >30 000 drug and gene perturbations, offering a huge repository of gene expression profiles, which can be browsed and compared to other data. We included DEGs from AMI‐specific WGCNA modules M7, M11, M14, M22, and M23, resulting in 268 upregulated and 125 downregulated genes useful for drug repurposing. Connectivity scores between changes of reference gene expression induced by drugs in THP1 cells and gene expression changes regarding the POSTEMI Large–Small comparison were calculated as described^[^ ^35^ ^]^. A score close to −1 indicates that the drug is likely able to reverse the gene expression signature, hence a promising candidate lead for therapeutic intervention. A full list of all candidates with connectivity scores between −1 and −0.60 as well as 0.60 and 1 and target information is available in Table S9 (Supporting Information).

### Statistics

Unless otherwise stated, data are presented as median ± 95% confidence interval (CI). For all Macroscreen functional data, each dot corresponds to the average of sample duplicates (discovery cohort) or triplicates (validation cohort). Prism v5.03 (GraphPad Software) was used for all statistical analyses. Normal distribution of data was tested with the D'Agostino–Pearson omnibus K2 test. Statistically significant differences were evaluated using Student's *t*‐test or the nonparametric Mann—Whitney *U* test as an alternative for not normally distributed data. Significant differences in binary data were analyzed with a Chi‐square test. For applications using computational methods, all Macroscreen datapoints were transformed to *z*‐scores, calculated separately for each functional read‐out parameter.

## Conflict of Interest

The authors declare no conflict of interest.

## Authorship Contributions

M.A.C.F., H.J., L.T., and E.A.L.B. contributed equally to this work. L.T., M.F., and E.B. designed the study. M.F. and L.T. performed the experiments. H.J. performed all computational analysis. M.G., N.A., and D.M. shared expert knowledge on high content screening. M.R. assisted with setting up of high content screening assays. E.W. performed RNA isolations and M.S. performed RNA sequencing experiments. L.S. and C.R. provided Annexin probes and expertise. C.S. provided multiplex ELISA equipment and expertise. L.G. and B.H. collected clinical samples for the validation cohort. G.A. and J.E. designed and collected samples for the POSTEMI cohort. P.H. did the CMR analyses for the POSTEMI cohort. M.S. and B.H. collected all control samples for discovery and validation cohort. P.A. and B.H. designed samples for the validation cohort and the matched control samples for the POSTEMI cohort. J.K. and E.S. gave critical advice on machine learning. D.M. and B.H. provided critical input to the manuscript. L.T., M.F., and H.J. wrote the manuscript. E.B. funded the study and critically reviewed the manuscript.

## Supporting information

Supporting Information

Supporting Information

Supporting Information

Supporting Information

Supporting Information

Supporting Information

Supporting Information

Supporting Information

Supporting Information

## Data Availability

The data that support the findings of this study are openly available in Gene Expression Omnibus at https://www.ncbi.nlm.nih.gov/geo/, reference number 172270.
