# Supplementary material for: Blood Milieu in Acute Myocardial Infarction Reprograms Human Macrophages for Trauma Repair
Source: Adv Sci (Weinh). 2022 Dec 16;10(5):2203053. doi: 10.1002/advs.202203053 (PMC9929255; doi:10.1002/advs.202203053)
Supplement: Supplementary file 1 — Supporting Information [file ADVS-10-2203053-s009.pdf]

ADVANCED SCIENCE
Open Access

Supporting Information

for *Adv. Sci.*, DOI 10.1002/advs.202203053

Blood Milieu in Acute Myocardial Infarction Reprograms Human Macrophages for Trauma Repair

Margaux A. C. Fontaine, Han Jin, Mick Gagliardi, Mat Rousch, Erwin Wijnands, Monika Stoll, Xiaofei Li, Leon Schurgers, Chris Reutelingsperger, Casper Schalkwijk, Nynke M. S. van den Akker, Daniel G.M. Molin, Lars Gullestad, Jan Eritsland, Pavel Hoffman, Mona Skjelland, Geir Ø. Andersen, Pål Aukrust, Joël Karel, Evgueni Smirnov, Bente Halvorsen, Lieve Temmerman and Erik A.L. Biessen*

Supplemental Material for

Blood milieu in acute myocardial infarction reprograms human macrophages for trauma repair

Authors: Margaux A. C. Fontaine[1,2]†, Han Jin[1,2]†, Mick Gagliardi[1,3], Mat Rousch[1,2], Erwin Wijnands[1,2], Monika Stoll[1,4], Xiaofei Li[2], Leon Schurgers[1,5], Chris Reutelingsperger[1,5], Casper Schalkwijk[1,6], Nynke M. S. van den Akker[1,3], Daniel G.M. Molin[1,3], Lars Gullestad[7,8], Jan Eritsland[9], Pavel Hoffman[9], Mona Skjelland[10,11], Geir Ø. Andersen[9], Pål Aukrust[10,12], Joël Karel[13], Evgueni Smirnov[13], Bente Halvorsen[10], Lieve Temmerman[1,2]*†, Erik A.L. Biessen[1,2,14]†.

† These authors contributed equally

Correspondence to: lieve.temmerman@mumc.nl

This file includes:

Supplemental Methods

Supplemental Figures S1 to S8

Supplemental Table S1

Supplemental References [1] – [5]

Other Supplemental Materials for this manuscript include the following:

Supplemental Tables S2 – S9

Supplemental Methods

Human von Willebrand Factor immunohistochemistry

Paraffin blocks containing cardiac tissue specimens obtained at autopsy from patients with acute myocardial infarction were derived from the pathology archives of the Maastricht University Medical Center, Maastricht. Criteria for the proper secondary use of human tissue in the Netherlands were met and consequently the MUMC Medical Ethical Board grants a waiver for the use of 'left-over materials' that are used anonymously. Blocks were cut into 3-µm-thick sections, deparaffinized and stained with ready-to-use rabbit polyclonal anti-human von Willebrand Factor antibody (Agilent DAKO, IR527). For the secondary antibody, polymer horseradish peroxidase (HRP) anti-rabbit antibody (Agilent DAKO) was used, and the immune complexes were detected using 3,3′-Diaminobenzidine (DAB, Agilent DAKO) as chromogen. Hematoxilin was used as nuclear counterstain. Pictures were taken on a Leica DM 3000 LED microscope at 10x magnification.

POSTEMI trial in- and exclusion criteria

The inclusion criteria in the POSTEMI trial[1] secured a rather homogenous STEMI population: Patients with first-time acute STEMI with symptom duration <6 h, referred to primary percutaneous coronary intervention (PCI), were considered for inclusion. ST-segment elevation in ECG >1 mm in at least 2 contiguous extremity leads or >2 mm in at least 2 precordial leads or new onset left bundle branch block had to be present. Clinically unstable patients with cardiac arrest, cardiogenic shock, hypotension, or pulmonary congestion were not eligible for inclusion. Patients with previous myocardial infarction, renal failure (serum creatinine >200 µmol/L), inability to provide informed consent, as well as patients with contraindications for CMR investigation were not included. All POSTEMI patients were treated with PCI.

Macroscreen functional profiling of stimulated macrophages (high content assays)

Lipid uptake: Fully differentiated 24 hrs stimulated macrophages were incubated for 2.5 hrs with a freshly prepared mix of Oxidized Low-Density-Lipoprotein (Ox-LDL, prepared as described before, 8 µg/mL[2]) and Topfluor (Avanti Polar Lipids, 2 µg/mL) in complete RPMI medium. Nuclei were stained with Hoechst 33342 (Sigma). Readout parameter is percentage of Topfluor-positive cells.

Phagocytosis: Fully differentiated 24 hrs stimulated macrophages were incubated for 1 h with pHrodo-labeled Zymosan (Thermo Fisher Scientific, 25 µL/mL) in complete RPMI medium

(100 µL). Nuclei were stained with Hoechst 33342 (Sigma). Readout parameter is percentage of pHrodo-positive cells.

Apoptosis: Fully differentiated 24 hrs stimulated macrophages were washed with Annexin Binding Buffer (10 mM HEPES, 140 mM NaCl, 5 mM $CaCl_2$; pH 7.4), and incubated with Annexin-V-OG (2.5 µg/mL) for 15 minutes.[3] Nuclei were stained with Hoechst 33342 (Sigma). Readout parameter is percentage of OG-positive cells.

Cell Shape: Fully differentiated 24 hrs stimulated macrophages were fixed and stained with Phalloidin 594 (Santa Cruz, binding F-actin) and Hoechst 33342 (Sigma, visualizing nuclei). Cell Profiler 3.1.8 software[4] was used to measure cell area (number of pixels within the segmented object), morphology (Form Factor, calculated as $4*\pi*\text{Area}/\text{Perimeter}^2$), actin stress fiber density (Phalloidin object intensity) and granularity (standard deviation of Phalloidin object intensity).

Inflammasome: Fully differentiated 24 hrs stimulated macrophages were primed with 0111:B4 (Invivogen, LPS 50 ng/ml) for 3 hrs, followed by 1 h incubation with Nigericin (Invivogen, 10 µM) at 37°C. Cells were fixed and stained overnight at 4°C with anti-ASC antibody (anti human ASC-PE, clone: TMS-1. Biolegend, 25µg/mL). Nuclei were visualized with Hoechst 33342 (Sigma). Readout parameter is percentage of PE-positive cells.

Efferocytosis: Jurkat T-cells P30 (ATCC TIB-152) were harvested in PBS+Mg/Ca (Gibco) at a concentration of 5 million cells/ml and labeled with calcein-AM (Invitrogen, 1mg/ml). After washing, staurosporin (ThermoFisher, 5 µM) was added in complete medium for 2 hrs at 37°C, 5% CO_2 to induce apoptosis. Fully differentiated 24 hrs stimulated macrophages were stained with Hoechst 33342 (visualizing nuclei, Sigma) and Phalloidin 594 (Santa Cruz, binding F-actin) and washed apoptotic Jurkats were added at a ratio of 3:1 in complete RPMI medium (100 µL) for 45 min at 37°C, 5% CO_2. Readout parameter is percentage of calcein-positive macrophages.

Multiplex ELISA cytokine measurement: After 24 hrs stimulation of macrophages with patient sera or control conditions, all medium was removed and fresh complete growth medium was added for another 8 hrs. Supernatant was snap-frozen and analyzed for levels of TNFα, IL8, IL6 and IL1β using V-plex human pro-inflammatory panel II cytokine ELISA (Mesoscale, MSD) assays, according to manufacturer's instructions.

Stimulation of macrophages

Patient sera were thawed and divided over 96-well copy plates stored at -80°C until use, ensuring a single freeze-thaw cycle for all subsequent analyses. Immediately before use, frozen sera copy plates were thawed at 37°C. Thawed sera were added to the growth medium of fully differentiated macrophages in a 1:5 dilution and incubated for 24 hrs at 37°C, 5% CO2. For Prostaglandin E2, interferon γ and interleukin 4 stimulation, fully differentiated macrophages were incubated with human recombinant PGE2 (Sigma, 1μg/ml), human recombinant IFNγ (Sigma, 200IU/ml) and human recombinant IL4 (Immunotools, 50ng/ml), respectively, in growth medium for 24 hrs at 37°C, 5% CO2.

RNA isolation from stimulated macrophages

Total RNA was isolated from 4-6 96-wells of 24 hrs AMI- or control discovery cohort sera stimulated human primary macrophages using the Micro RNeasy kit (Qiagen) following the <100 000 cells protocol. 3 AMI samples were excluded from further analysis due to low RNA concentration, meaning a total of 47 AMI and 20 control exposed samples were included in the transcriptional analysis. Of those 47 AMI samples, 23 were obtained from the POSTEMI-Small group, 24 from the POSTEMI-Large group. For the PGE2-, IFNγ- and IL4- stimulated and corresponding control macrophages, 3 independent replicates for each condition were included. RNA quality and integrity were assessed on a 2100 BioAnalyzer Picochip (Agilent Technologies). Average RIN value was 7.5 (95% confidence interval 7.3-7.7).

RNA Sequencing

Next generation RNA Sequencing was performed on isolated RNA from 24 hrs AMI- or control sera stimulated macrophages. 24 hrs PGE2-, IFNγ- and IL4- stimulated macrophages and control RNA samples (n=3) were processed in a separate run using an identical sequencing protocol. Quality controlled total RNA (Agilent Bioanalyzer RIN value >7) was enriched for the Poly(A) RNA fraction by use of the NEBNext Poly(A) mRNA Magnetic Isolation Modul (NEB). Subsequent cDNA library preparation was carried out using the NEBNExt Ultra II Directional RNA Library Prep Kit for Illumina (NEB) according to the manufacturer´s instructions. The size of the resulting library was checked (Agilent Tape Station) and quantified by qPCR (NEBNExt Library Quant Kit for Illumina, NEB). Equimolar pooled libraries were sequenced in a single read mode (75 cycles) on a NextSeq500 system (Illumina) and v2.5 chemistry at a depth of about 10M reads per sample.

RNA Sequencing data preprocessing and quality control

Quality of raw sequences of macrophages treated by patients' sera (including control) was inspected with FastQC (v0.11.8). All data passed quality control. All sequencing data were aligned to the reference human genome sequence GRCh38 primary assembly version and its corresponding annotation on GTF file (https://www.gencodegenes.org/human/) using STAR (v2.4.0.1). Gene expression from aligned sequences to read counts was quantified using HTSeq (v0.11.1). The average rate of successfully uniquely mapped reads was 90.3%. Low-expressed genes with <10 counts in > 90% of the samples were removed, leaving 12,341 genes for downstream analyses. Normalization and variance-stabilizing transformation on read counts were carried out by DESeq2 package (v1.24.0) in R. The R package sva (v3.34.0) was used to ensure no batch effects influenced data output. RNA-seq data of untreated and IL4- IFNγ-, and PGE2-treated macrophages were processed in a similar manner. In particular, genes with an average count < 5 were removed from the analysis.

Computational methods

Sample Distribution: Principal Component Analysis (PCA) was deployed on variance-stabilizing transformed gene expression data of the discovery cohort. All samples were plotted on the first two principal components. Explained variance was calculated to reflect how much total variance can be represented by each principal component. Similarly, PCA was deployed on gene expression data from the GSE59867 heart failure (n=9) and non-heart failure (n=8) samples at time point 6h, as well as the untreated (M0), IL4-treated (M2) and IFNγ-treated (M1) macrophages. Batch effects were corrected by the function removebatcheffect provided in limma (v3.50.3) R package.

Differential gene expression analysis: Raw read counts without normalization from the complete Discovery cohort and the PGE2-stimulated versus control macrophages RNA sequencing datasets were given as input in DESeq2 to detect differentially expressed genes (DEGs). Between AMI-mac and Ctrl-mac, the adjusted p-value cutoff was set at < 0.05 and absolute log2 fold change cutoff at > 1 (positive log2 fold change means higher expression in AMI, vice versa; see Table S1). Between POSTEMI-Small and POSTEMI-Large samples, the p-value cutoff was set at < 0.05 (positive log2 fold change means higher expression in POSTEMI-Large, vice versa; see Table S2). Between PGE2-stimulated and control macrophages, the adjusted p-value cutoff was set at < 0.05.

Supplemental Figures

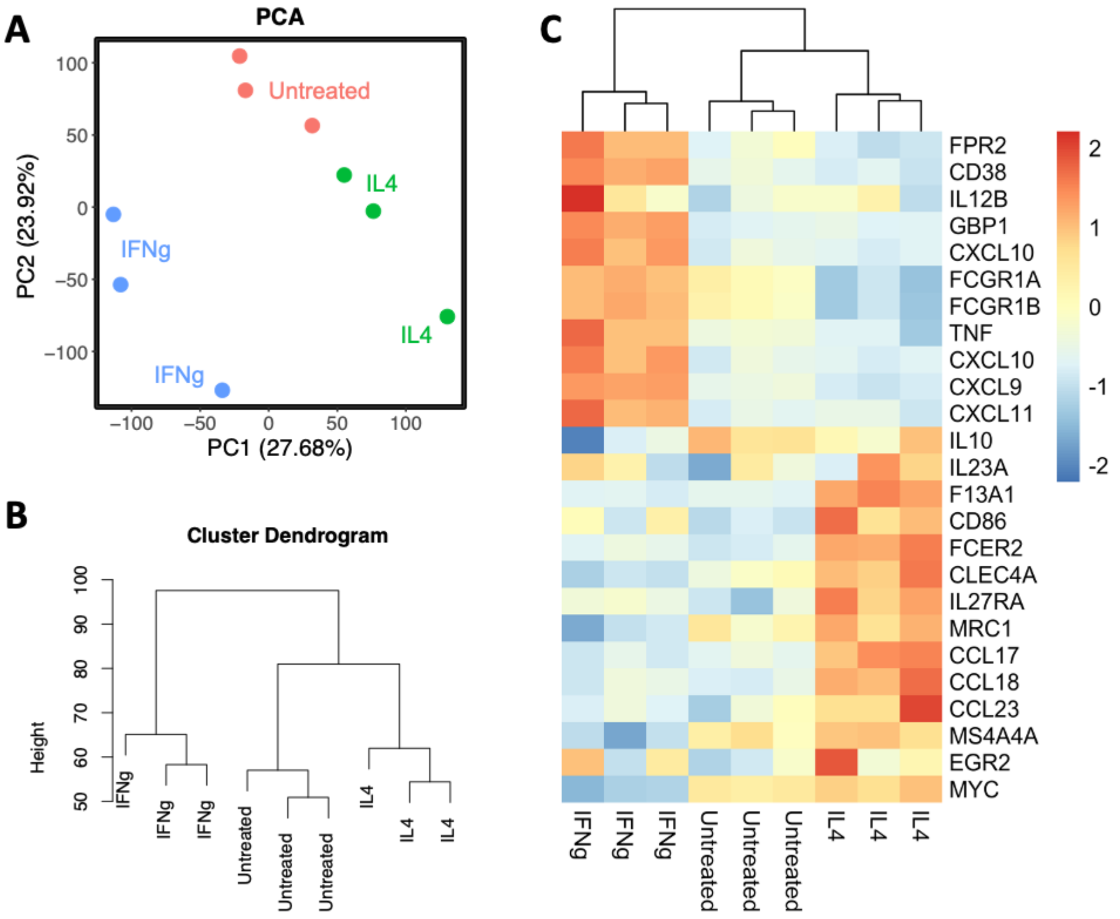

Figure S1: Characterization of human CD14+ monocyte-derived macrophages

(A) Principal component analysis (PCA) plot showing the distribution of M0 (untreated), M1 (IFNg-treated), and M2 (IL4-treated) macrophages (*n*=3) based on gene expression profile (B) Hierarchical clustering based on the gene expression profile of the M0, M1, and M2 macrophages (*n*=3) (C) Heatmap showing the expression of signature macrophage markers with the samples hierarchically clustered. (*n*=3)

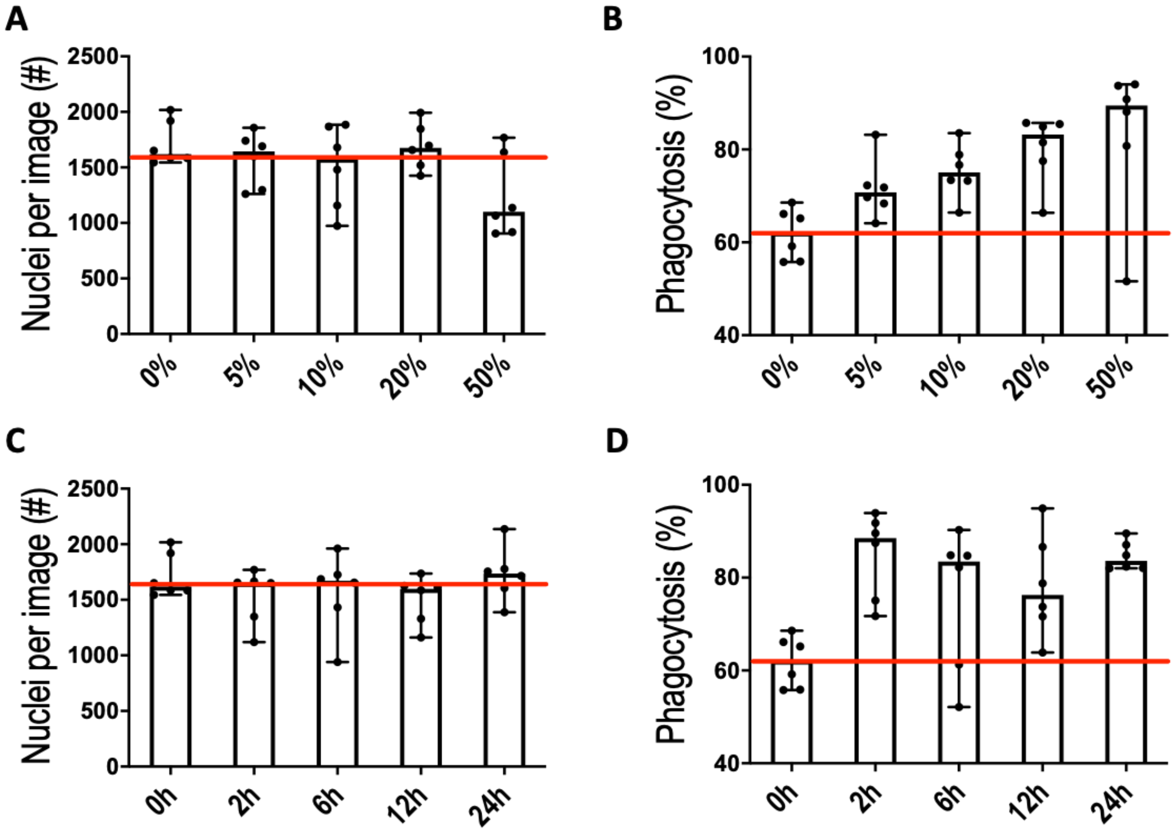

Figure S2. Determination of optimal serum incubation conditions

Human primary macrophages were incubated for 24 hrs with increasing percentages (A, B) or different timepoints of 20% (C, D) of pooled serum. *n*=6 pools of 3 serum samples each. (A, C) Counts of Hoechst-labeled nuclei. (B, D) Phagocytosis of pHrodo-labeled Zymosan particles.

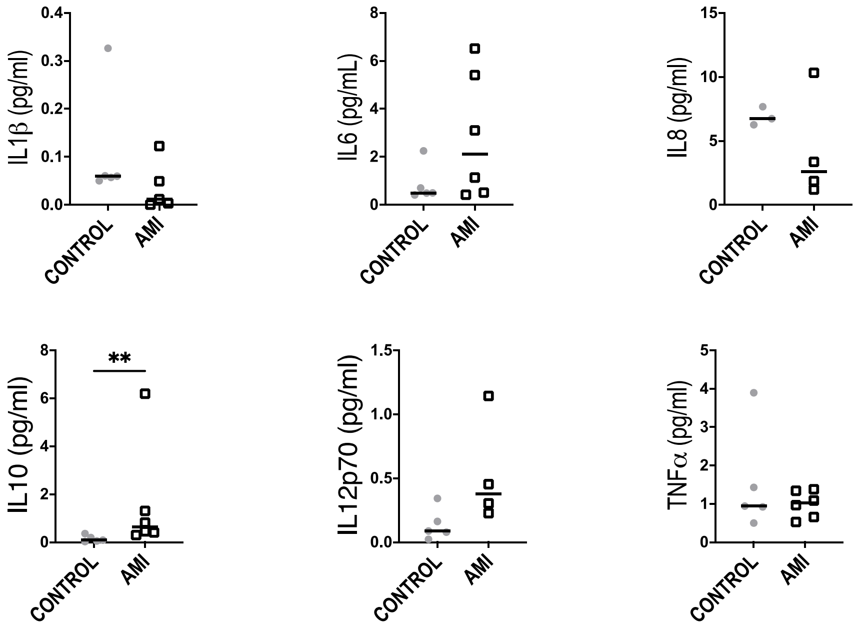

Figure S3. Cytokine content of human serum

MSD multiplex ELISA measurements on human serum used for macrophage incubations. 4-6 biological replicates were pooled to obtain 1 data point. *n*=5 for controls, *n*=6 for AMI. Below detection values are not shown.

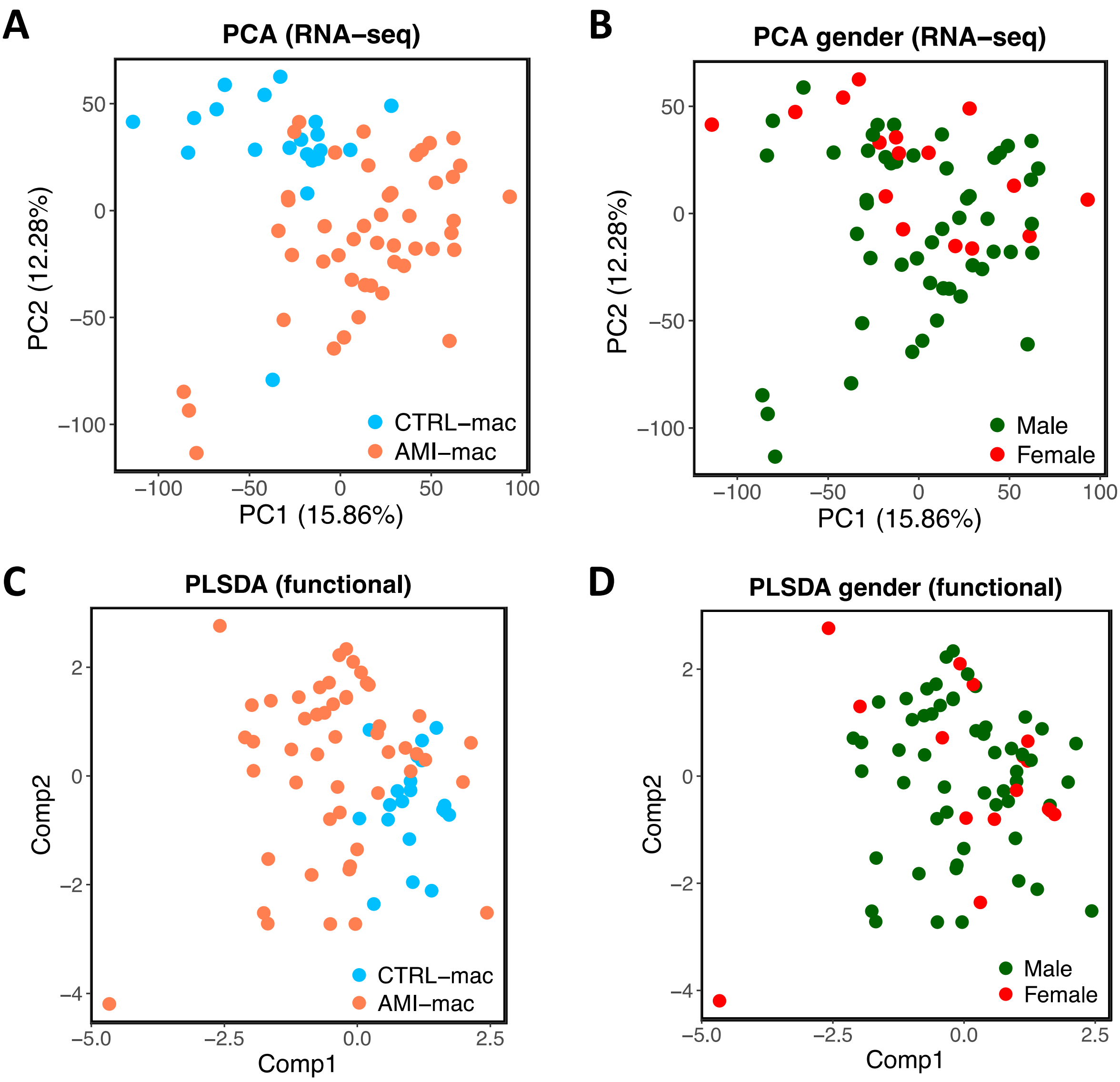

Figure S4: Gender is not a confounder in the AMI- versus Ctrl-mac segregation

(A) Principal component analysis (PCA) plot showing the distribution of AMI- and Ctrl-mac based on gene expression profile. (B) PCA plot of the same AMI- and Ctrl-mac distribution as shown in A, color-coded for gender. (C) Partial least squares-discriminant analysis (PLS-DA) showing the classification of AMI- and Ctrl-mac based on Macroscreen functional profiling data. (D) PLS-DA plot of the same AMI- and Ctrl-mac distribution as shown in C, color-coded for gender.

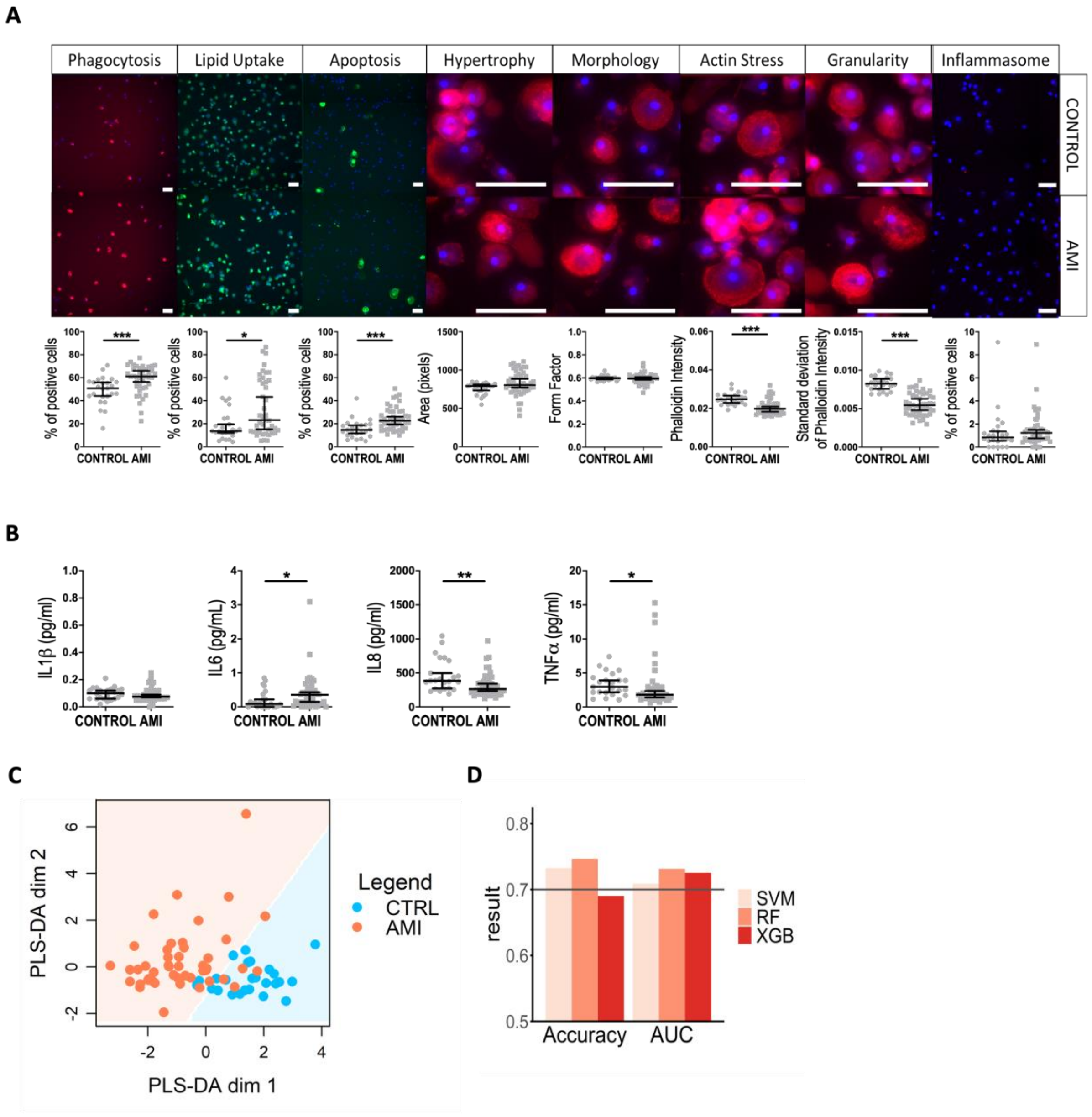

Figure S5. Functional signature in primary human macrophages induced by independent validation AMI serum cohort

(A) High content screening images with corresponding image cytometric data graphs for phagocytosis, lipid uptake, apoptosis, hypertrophy#, morphology#, actin stress#, granularity# and inflammasome## assays of AMI- mac versus Ctrl-mac with 10x, #40x, or ##20x magnification, respectively. Scalebar is 20 µm. (B) Macrophage cytokine secretion after 24 hrs AMI or control serum exposure. (C) PLS-DA based on Macroscreen data. (D) ROC AUC and Accuracy (Mean ± SD) in SVM, RF and XGB Discovery cohort classification applied on Macroscreen data from the Validation cohort. n=47 AMI, n=25 ctrl. * $p<0.05$, ** $p<0.01$, *** $p<0.001$.

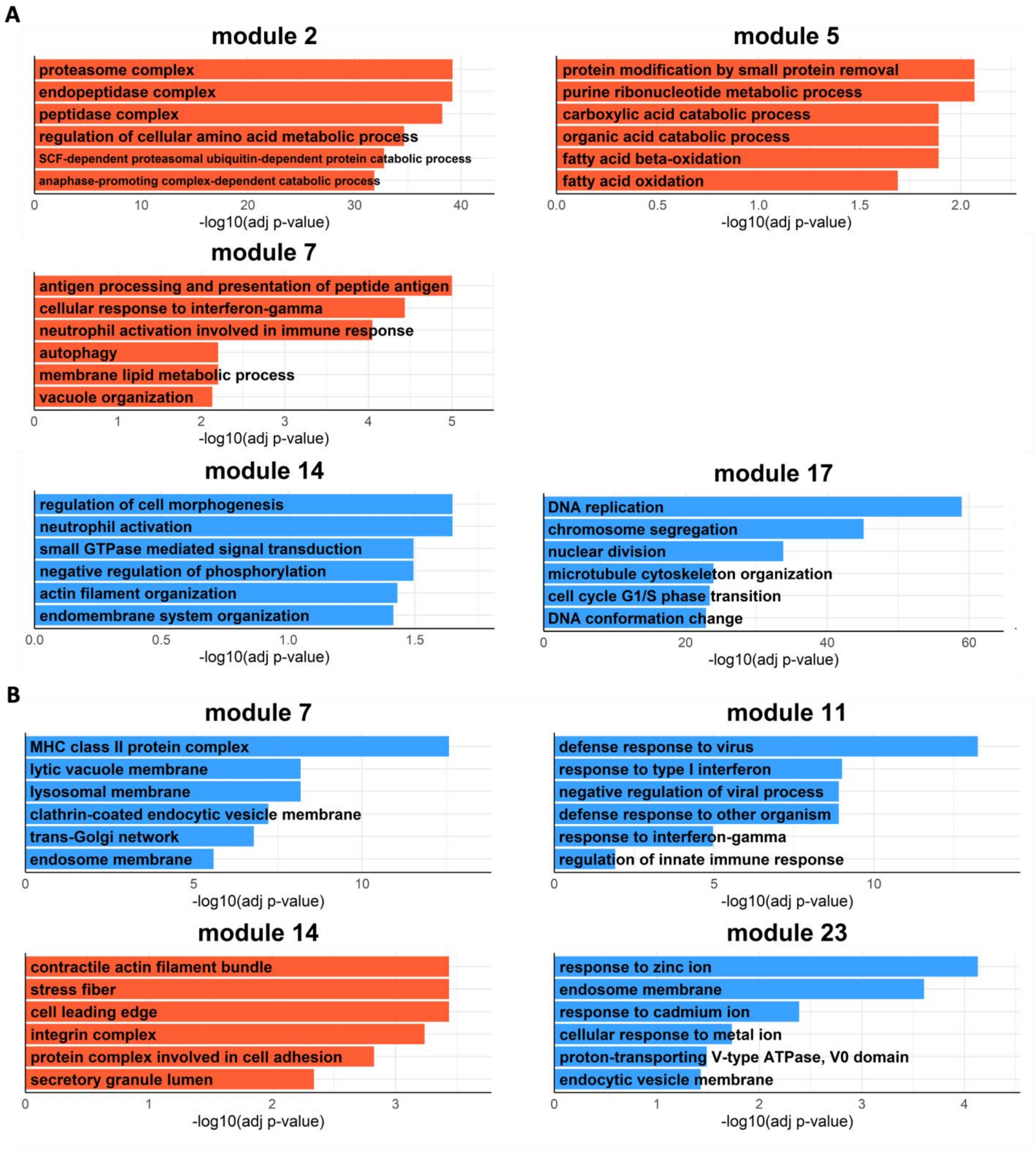

Figure S6. Gene Ontology terms associated with AMI-serum reprogramming

(A) Gene Ontology Enrichment Analysis (GOEA) graphs of AMI-mac and Ctrl-mac WGCNA gene modules. (B) GOEA graphs of AMI-specific WGCNA gene modules.

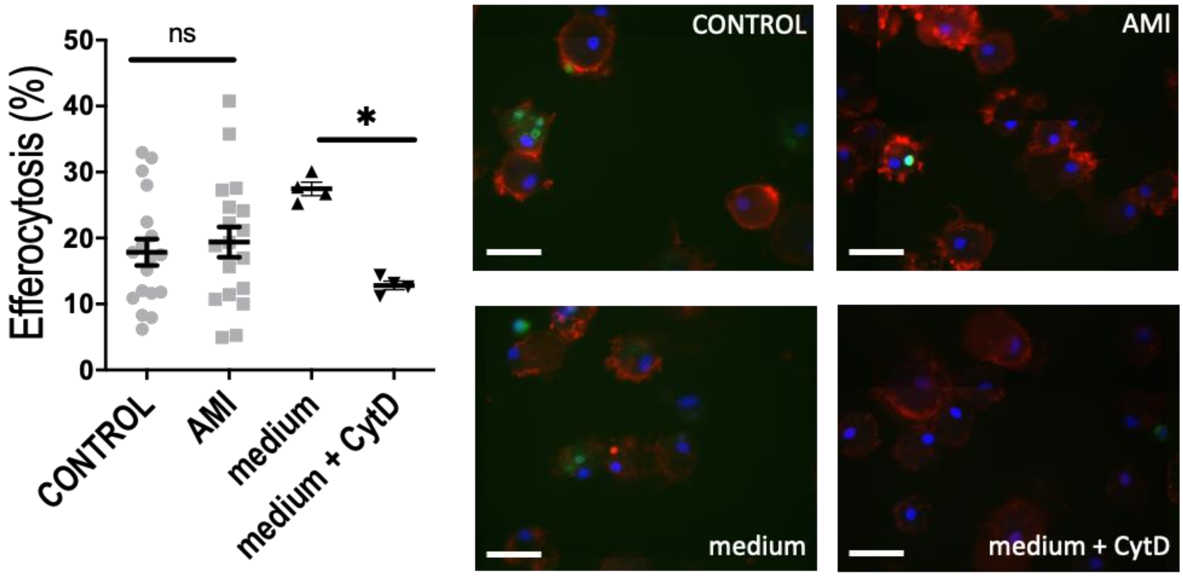

Figure S7. AMI serum incubation has no effect on efferocytosis capacity of human macrophages

Human primary macrophages were incubated for 24hrs with serum (Control or AMI), left in growth medium, or treated with Cytochalasin D. Macrophages were stained with Phalloidin (actin, red) and Hoechst (nuclei, blue) and Calcein-labeled apoptotic Jurkats (green) were added. Efferocytosis was assessed as % of Calcein-positive macrophages. *: $p<0.05$. Scalebar is 10 µm.

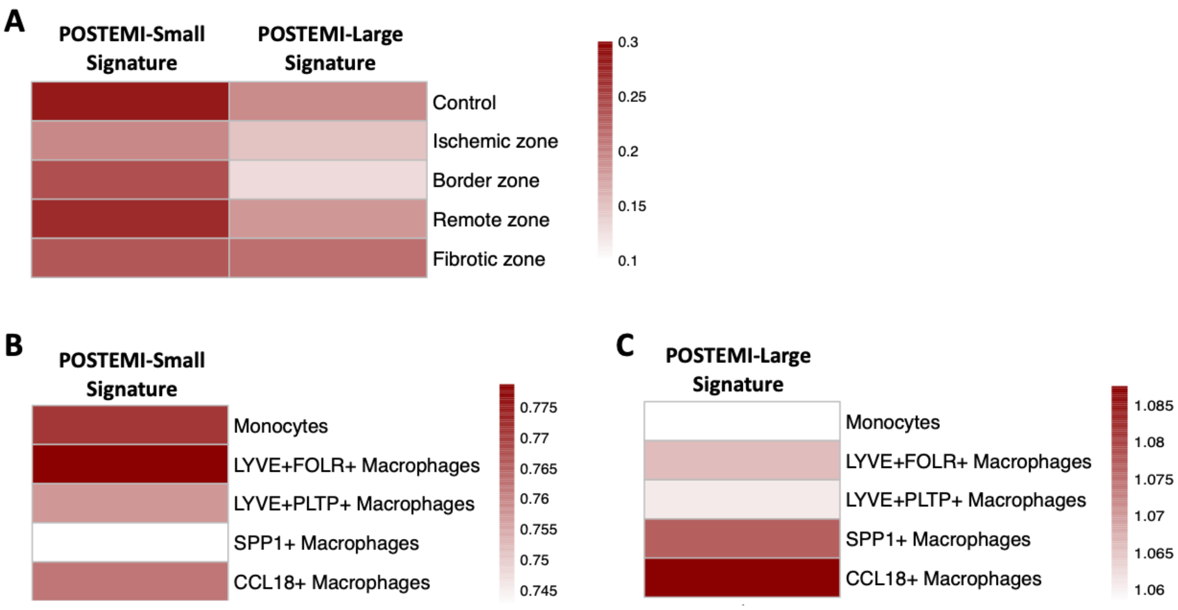

Figure S8: Serum-induced macrophage genes are enriched in human infarcted hearts

Single Sample GeneSet Enrichment Analysis (ssGSEA) of scRNASeq data from the myeloid cell populations of human infarcted hearts.[5] (A) Heatmap shows the relative enrichment scores of POSTEMI-Small and POSTEMI-Large specific DEGs in the myeloid cells within the different areas of the heart. (B, C) Heatmap shows the relative enrichment scores of POSTEMI-Small (B) and POSTEMI-Large (C) specific DEGs in identified cardiac macrophage subtypes.

Supplemental Tables

Table S1. WGCNA module correlation to gender

	Correlation to AMI	p-value	Correlation to gender	p-value
**Module 2**	0.63	1e-08	0.24	ns
**Module 5**	0.62	2e-08	0.11	ns
**Module 7**	0.7	5e-11	0.22	ns
**Module 14**	-0.51	8e-06	-0.13	ns
**Module 17**	-0.71	1e-11	-0.35	0.0038

Pearson correlation coefficient (PCCs) between AMI-specific WGCNA modules, and the AMI phenotype and gender respectively, with corresponding p-values.

Supplemental References

[1] S. Limalanathan, G. O. Andersen, P. Hoffmann, N. E. Klow, M. Abdelnoor, J. Eritsland, *Cardiology* **2010**, *116* (2), 103, https://doi.org/10.1159/000316965.

[2] W. L. Hendriks, H. van der Boom, L. C. van Vark, L. M. Havekes, *Biochem J* **1996**, *314 (Pt 2)*, 563, https://doi.org/10.1042/bj3140563.

[3] I. Vermes, C. Haanen, H. Steffens-Nakken, C. Reutelingsperger, *J Immunol Methods* **1995**, *184* (1), 39, https://doi.org/10.1016/0022-1759(95)00072-i.

[4] A. E. Carpenter, T. R. Jones, M. R. Lamprecht, C. Clarke, I. H. Kang, O. Friman, D. A. Guertin, J. H. Chang, R. A. Lindquist, J. Moffat, P. Golland, D. M. Sabatini, *Genome Biol* **2006**, *7* (10), R100, https://doi.org/10.1186/gb-2006-7-10-r100.

[5] C. Kuppe, R. O. Ramirez Flores, Z. Li, S. Hayat, R. T. Levinson, X. Liao, M. T. Hannani, J. Tanevski, F. Wunnemann, J. S. Nagai, M. Halder, D. Schumacher, S. Menzel, G. Schafer, K. Hoeft, M. Cheng, S. Ziegler, X. Zhang, F. Peisker, N. Kaesler, T. Saritas, Y. Xu, A. Kassner, J. Gummert, M. Morshuis, J. Amrute, R. J. A. Veltrop, P. Boor, K. Klingel, L. W. Van Laake, A. Vink, R. M. Hoogenboezem, E. M. J. Bindels, L. Schurgers, S. Sattler, D. Schapiro, R. K. Schneider, K. Lavine, H. Milting, I. G. Costa, J. Saez-Rodriguez, R. Kramann, *Nature* **2022**, *608* (7924), 766, https://doi.org/10.1038/s41586-022-05060-x.
